# Homeodomain protein PRRX1 anchors the Ku heterodimers at DNA double-strand breaks to promote nonhomologous end-joining

**DOI:** 10.1093/nar/gkaf200

**Published:** 2025-03-20

**Authors:** Yan Wang, Fuyuan Shen, Chen Zhao, Jiali Li, Wen Wang, Yamu Li, Jia Gan, Haojian Zhang, Xuefeng Chen, Qiang Chen, Fangyu Wang, Ying Liu, Yan Zhou

**Affiliations:** Department of Neurosurgery, Medical Research Institute, Zhongnan Hospital of Wuhan University, Wuhan University, Wuhan 430071, China; Frontier Science Center of Immunology and Metabolism, Wuhan University, Wuhan 430071, China; Department of Neurosurgery, Medical Research Institute, Zhongnan Hospital of Wuhan University, Wuhan University, Wuhan 430071, China; Frontier Science Center of Immunology and Metabolism, Wuhan University, Wuhan 430071, China; Department of Neurosurgery, Medical Research Institute, Zhongnan Hospital of Wuhan University, Wuhan University, Wuhan 430071, China; Frontier Science Center of Immunology and Metabolism, Wuhan University, Wuhan 430071, China; Department of Neurosurgery, Medical Research Institute, Zhongnan Hospital of Wuhan University, Wuhan University, Wuhan 430071, China; Frontier Science Center of Immunology and Metabolism, Wuhan University, Wuhan 430071, China; Department of Neurosurgery, Medical Research Institute, Zhongnan Hospital of Wuhan University, Wuhan University, Wuhan 430071, China; Frontier Science Center of Immunology and Metabolism, Wuhan University, Wuhan 430071, China; The First Affiliated Hospital of Henan University, Kaifeng 475004, China; Department of Neurosurgery, Medical Research Institute, Zhongnan Hospital of Wuhan University, Wuhan University, Wuhan 430071, China; Frontier Science Center of Immunology and Metabolism, Wuhan University, Wuhan 430071, China; Frontier Science Center of Immunology and Metabolism, Wuhan University, Wuhan 430071, China; Frontier Science Center of Immunology and Metabolism, Wuhan University, Wuhan 430071, China; Frontier Science Center of Immunology and Metabolism, Wuhan University, Wuhan 430071, China; Department of Neurosurgery, Medical Research Institute, Zhongnan Hospital of Wuhan University, Wuhan University, Wuhan 430071, China; Frontier Science Center of Immunology and Metabolism, Wuhan University, Wuhan 430071, China; Department of Neurosurgery, Medical Research Institute, Zhongnan Hospital of Wuhan University, Wuhan University, Wuhan 430071, China; Frontier Science Center of Immunology and Metabolism, Wuhan University, Wuhan 430071, China; Department of Neurosurgery, Medical Research Institute, Zhongnan Hospital of Wuhan University, Wuhan University, Wuhan 430071, China; Frontier Science Center of Immunology and Metabolism, Wuhan University, Wuhan 430071, China

## Abstract

The DNA-dependent protein kinase (DNA–PK) complex plays a critical role in nonhomologous end-joining (NHEJ), a template-independent pathway for repairing DNA double-strand breaks (DSBs). The association of Ku70/80 with DSB ends facilitates the assembly of the DNA–PK holoenzyme. However, key mechanisms underlying the attachment and stabilization of DNA–PK at broken DNA ends remain unclear. Here, we identify PRRX1, a homeodomain-containing protein, as a mediator of chromatin localization and subsequent activation of DNA–PK. PRRX1 oligomerizes to simultaneously bind to double-strand DNA and the SAP (SAF-A/B, Acinus, and PIAS) domain of Ku70, thereby enhancing Ku anchoring at DSBs and stabilizing DNA–PK for efficient NHEJ repair. Reduced expression or pathogenic mutations of PRRX1 are associated with genomic instability and impaired NHEJ repair. Furthermore, a peptide that disrupts PRRX1 oligomerization compromises NHEJ efficiency and reduces cell survival following irradiation. These findings provide new insights into the activation of the NHEJ machinery and offer potential strategies for optimizing cancer therapies.

## Graphical Abstract

**Graphical Abstract:**
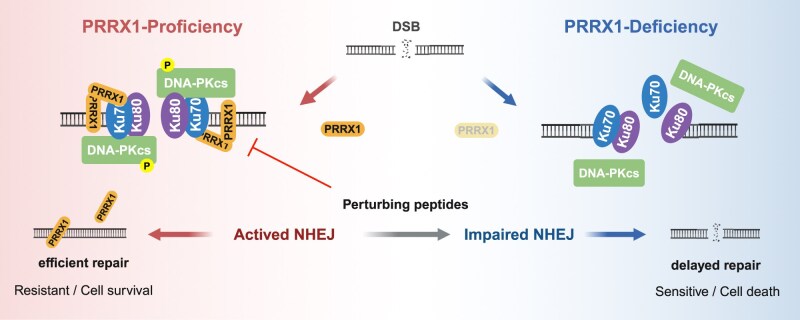

## Introduction

DNA double-strand breaks (DSBs) are among the most deleterious genomic lesions in mammalian cells [1–3]. DSBs are primarily repaired through two classical pathways [4–6]: nonhomologous end-joining (NHEJ) and homologous recombination (HR). HR utilizes homologous DNA sequences as a template, making it a high-fidelity repair mechanism favored during the S/G2 phase of the cell cycle [7, 8]. In contrast, NHEJ is a rapid but error-prone DNA repair process that directly rejoins broken DNA strands without requiring a template and remains active throughout the cell cycle [9, 10]. Overactivation of DNA damage repair (DDR) mechanisms, including NHEJ, is commonly observed in cancer cells, contributing to resistance against radiotherapy and chemotherapy. This presents a significant challenge in cancer treatment that demands urgent attention [11, 12].

In NHEJ, DSB ends are initially recognized and bound by the Ku70/80 heterodimer, which, acting as a scaffold, recruits the catalytic subunit of DNA-dependent protein kinase (DNA-PKcs) to form the DNA–PK complex. Subsequently, DNA–PK is activated by DNA ends to initiate the repair [13, 14]. The crystal structure of the Ku heterodimer reveals that each Ku70/80 subunit consists of three distinct regions: the N-terminal von Willebrand A domain, a β-barrel domain associated with dimerization, and the C-terminal domain [15, 16]. The C-terminal extension of Ku80 contributes to the stabilization of the DNA–PK complex [17, 18], while the SAP (SAF-A/B, Acinus, and PIAS) domain of Ku70 C-terminal is essential for the stable association of the Ku70/80 heterodimer with double-stranded DNA (dsDNA) ends *in vitro* [19–21]. The Ku70/80 heterodimer exhibits high affinity with broken DNA ends, independent of the DNA sequences, enabling its prompt translocation onto DSB sites. Additionally, Ku70/80 can bind directly or indirectly to various histone modifications, facilitating its recruitment to DSB-associated chromatin [22, 23]. Furthermore, nuclear factors such as MRI/CYREN [24, 25], LRF [26], BRN2 [27], ZNF384 [28], and SMYD2 [29], have been implicated in regulating Ku dynamics during NHEJ repair. These findings underscore the complexicity of Ku dynamics. However, critical question remains regarding how the attachment and stabilization of Ku–DNA–PKcs complex are precisely regulated on the chromatin context. Unraveling these mechanisms is essential for a comprehensive understanding of how sensor proteins respond to DSBs and initiate NHEJ.

PRRX1, a member of the PRD class of homeodomain (HD) protein family, plays numerous roles in regulating skeletal cartilage development, epithelial–mesenchymal transition, fibrosis, and other mesoblast lineage-related biological processes [30–33]. Its primary function is mediated through DNA-binding activity, either as a transcription factor or co-activator. A recent study has showed that PRRX1, in conjunction with FOXM1, promotes the expression of HR-associated genes in prostate cells [34]. In this study, we unexpectedly revealed a transcription-independent role of PRRX1 in promoting NHEJ. We found that oligomerized PRRX1 directly associates with both Ku and dsDNA to facilitate the assembly and activation of DNA–PK at DSBs. Moreover, pathogenic mutations and low expression of PRRX1 in stomach adenocarcinomas (STADs) are linked to genomic instability and defective NHEJ. Notably, we demonstrated that the peptide disrupting PRRX1 oligomerization impairs NHEJ efficiency and reduces cell survival following irradiation. This study not only provides novel insight into the role of HD protein in regulating NHEJ, but also holds implications for the development of cancer treatment strategies.

## Materials and methods

### Animals and genotyping

All animal procedures were approved by the Animal Care and Ethical Committee of Medical Research Institute at Wuhan University. *Prrx1*^+/−^ C57BL/6JGpt mice (Strain No. T013725; RRID: IMSR_GPT:T013725) were generated by the GemPharmatech (Nanjing, China) using the CRISPR/Cas9-mediated gene editing. Two single-guide RNAs (sgRNAs) were designed to generate a chromosomal deletion at the second exon region of *Prrx1* in the mouse genome. Targeting vector, Cas9 vector, and sgRNAs were microinjected into mouse zygotes. Zygotes were transferred into pseudo-pregnant female mice to generate founders, which were genotyped by polymerase chain reaction (PCR) and sequencing. Positive founders were crossed with C57BL/6 wild-type (WT) mice to generate F1 mice. The primer set for genotyping is provided in Supplementary Table S3. Band sizes for the WT allele and knockout (KO) allele are 614 and 542 bp, respectively.

### Cell cultures

HEK293T (RRID: CVCL_0063) cell line was a gift from Dr Hongbing Shu (Wuhan University). MOVAS (RRID: CVCL_0F08) cell line was a gift from Dr Xiang Cheng (Union Hospital, Huazhong University of Science and Technology). WT (CHO-AA8, RRID: CVCL_4386) and *Xrrc6*^−/−^ (CHO-xrs-6, RRID: CVCL_4340) cell lines were gifts from Dr Dongyi Xu (Peking University). EJ5 U2OS cell line was a gift from Dr Jun Huang (Zhejiang University). U2OS (RRID: CVCL_0042) and Hs746.T (RRID: CVCL_0333) cell lines were purchased from CCTCC. Mouse embryonic fibroblasts (MEFs) were generated from WT or E14.5 *Prrx1*^−^*^/^*^−^ C57BL/6 littermates. *Trp53bp1*^−/−^ MEF (RRID: CVCL_UJ19) cell line was a gift from Dr Qiang Chen (Wuhan University). U2OS cells were cultured in McCoy’s 5A (Pricella, PM150710) and others were cultured in Dulbecco’s modified Eagle’s medium (Gibco, C11995500CP) containing 10% fetal bovine serum (NEWZERUM, FBS-S500) and 1% penicillin–streptomycin (10 000 U/ml; Gibco, 15140-122) at 37°C in 5% (v/v) CO_2_.

### Constructs, antibodies, and reagents

The complementary DNAs (cDNAs) of PRRX1A (PA) and PRRX1B (PB) were subcloned into the pEGFP-C1, pmCherry-C1, pHAGE-zsgreen/puro, pMAL-c2X, and pET-28a (+) vectors for expression of N-terminus-tagged or C-terminus-tagged fusion proteins. The cDNAs of Ku70 and Ku80 were cloned into pGEX-4T-1 for recombinant protein expression and purification. All deletion and point mutants used in this study were generated by PCR and confirmed by DNA sequencing.

Primers for constructs and antibodies used in the study are listed in Supplementary Table S3. Etoposide (ETP; E1383), camptothecin (CPT; C9911), Thymidine (T1895), and Polybrene (TR-1003) were purchased from Sigma–Aldrich (RRID: SCR_008988). Puromycin (S7417), KU55933 (S1092), NU7441 (S2638), and Olaparib (S1060) were purchased from Selleckchem (RRID: SCR_003823). Mirin (HY-117693) was purchased from MedChemExpress (RRID: SCR_025062).

### shRNAs, lentivirus production and cell transfection

For transient expression, cells were transfected with expression vectors using Polyethylenimine (PEI) (Sigma, 919012) according to the manufacturer’s instructions. Short-hairpin RNAs (shRNAs) targeting human *XRCC6* (Ku70) and a nontargeting control (Scramble) were cloned into pLKO.1 vector for stable expression. Lentiviral-based constructs were co-transfected with packaging plasmids pMD2.G and psPAX2 into HEK293T cells for subsequent harvest and stable gene expression. U2OS and Hs746T cells were transduced with lentivirus encoding shRNAs in the presence of 5 μg/ml Polybrene (1000×) and selected with 2.5 μg/ml puromycin. Target-sequence information of the shRNAs was listed in Supplementary Table S3.

### Modified DNA substrates

All oligos shown in Supplementary Table S3 for DNA pull-down or microscale thermophoresis (MST) assay were synthesized by Sangon Biotech. The blunt and overhang dsDNA (biotinylated or 6-FAM-labelled) were prepared by annealing complementary oligos in a PCR machine (Bio-Rad C-1000 Thermal Cycler; RRID: SCR_019688): heating for 3 min to 95°C, then gradually cooling over a period of 45 min to room temperature (RT).

For DNA pull-down assay, Oligo#2-S was used as the single-stranded DNA (ssDNA) substrate, while the blunt dsDNA was formed by annealing Oligo#1-S with Oligo#1-A. The 3′ overhang DNA was formed by annealing Oligo#1-A with Oligo#2-S, and 5′ overhang DNA ormed by annealing Oligo#1-S with Oligo#2-A. In the MST assay, the blunt dsDNA was formed by annealing Oligo#3-S with Oligo#1-A, while dsDNA with various lengths of 5′ overhang was generated by annealing Oligo#3-S with Oligo#4-A, Oligo#5-A, or Oligo#6-A.

### Peptides

All peptides were synthetized by GenScript (RRID:SCR_002891; Nanjing, China). Synthetic peptides were purified to >95% purity by high-pressure liquid chromatography. Peptides were dissolved in ultrapure water to generate a 10 μg/μl stock solution and worked at a moderate concentration (40 μM) for experiments in cell. The peptides contain biotin and a TAT sequence at their N-termini as follows: competing peptide, biotin-GRKKRRQRRR-QALERVFER; mutated peptide, biotin-GRKKRRQRRR-AALAAVFAA.

### Irradiation, laser micro-irradiation, and live imaging

Ionizing radiation (IR) was delivered to cells using an X-ray generator machine (160 KV, 25 mA, 1.2 Gy/min, Rad Source RS2000Prox). MOVAS, U2OS, and Hs746T cells were seeded on glass-bottom dishes (NEST, 801001) and transfected with the indicated plasmid using PEI 24 h prior to irradiation. Laser micro-irradiation (LMI) was performed using the MicroPoint Laser Illumination and Ablation System (Andor iQ; RRID: SCR_014461) with a 337 nm laser (3.4 mW) generating a 365 nm UVA laser to induce DNA breaks in nuclei. Cells were either imaged live or fixed with 4% paraformaldehyde (PFA) for antibody staining. Images were captured using an Olympus IX71 microscope (RRID: SCR_022185). GFP fluorescence at the laser stripe was analyzed with ImageJ, and the relative intensity (background-to-stripe ratio) was calculated for each timepoint and expressed as mean ± standard error of the mean (SEM).

### Immunofluorescence

Cells were cultured on coverslips prior to experiments. For γH2AX staining, cells were fixed with 4% PFA at RT for 15 min and washed twice with phosphate-buffered saline (PBS). For 53BP1 and pS2056 DNA-PKcs staining, cells were pre-extracted on ice for 5 min using pre-extraction buffer [20 mM HEPES (pH 7.4) 20 mM NaCl, 5 mM MgCl_2_, 0.5% NP-40, 1 mM Dithiothreitol (DTT), protease inhibitor cocktail], followed by fixation with 4% PFA and blocking at RT for 30 min. Primary antibodies were diluted in Tris Buffered Saline with Tween 20 (TBST) with 5% bovine serum albumin (BSA) and incubated overnight at 4°C. After washing, secondary antibodies diluted in blocking buffer were applied for 1 h at RT. Cells were washed twice and stained with DAPI (1:1000 in PBS) or PicoGreen (1:500 in PBS) for 5 min at RT. After final washes, cells were mounted with antifade solution and imaged using a Leica Stellaris 5 WLL confocal microscope (RRID: SCR_024663). All washes were performed in PBS, and blocking was done in NGS buffer [3% normal goat serum, 10 mM Tris–HCl (pH 7.4), 100 mM NaCl, 0.1% Triton X-100, 0.1% BSA].

### Affinity purification and mass spectrometry

Glioma-initiating cells (GICs) stably overexpressing FLAG-PA or FLAG-PB (the bait) were lysed with RIPA buffer [20 mM Tris–HCl (pH 7.5), 150 mM NaCl, 1 mM ethylenediaminetetraacetic acid (EDTA), 1 mM DTT, 0.5% sodium deoxycholate, 1% NP-40] on ice for 20 min. U2OS cells transiently overexpressing FLAG-PA were lysed using ice-cold lysis buffer [10 mM HEPES–NaOH (pH 8.0), 10 mM KCl, 1.5 mM MgCl_2_, 0.5 mM β-mercaptoethanol] supplemented with protease inhibitor for 15 min, followed by the addition of 0.2% NP-40 for 2 min. After centrifugation (16 000 × *g* for 10 min at 4°C; Eppendorf 5810R; RRID: SCR_019855), pellets were washed twice with cold PBS and resuspended in RIPA buffer. Each lysate was centrifuged again at 16 000 × *g* for 15 min, and the supernatants were incubated with anti-FLAG affinity agarose gel (Bimake, B23012) overnight at 4°C. The resin was washed five times with lysis buffer, and the proteins bound to the beads were eluted at 95°C for 5 min with sodium dodecyl sulfate (SDS) loading buffer. Eluted proteins were identified by mass spectrometry (MS). Each bait was processed independently and analyzed alongside empty vector (EV) negative controls (cells expressing no bait), which were grown in parallel and treated identically to the bait-expressing cells.

### Immunoblotting and immunoprecipitation

For immunoblotting (IB), cells were directly lysed with 1× SDS loading buffer [50 mM Tris–HCl (pH 6.8), 10% glycerol, 2% SDS, 0.01% bromophenol blue, 1% β-mercaptoethanol] and heated at 95°C for 10 min. The supernatants were then subjected to a sodium dodecyl sulfate–polyacrylamide gel electrophoresis (SDS–PAGE) and immunoblotted using the indicated antibodies (primary antibody: diluted in 5% BSA TBST buffer; secondary antibody: diluted in 5% milk TBST buffer). Protein signals were detected using Western ECL Substrate (Bio-Rad) and captured on medical X-ray film.

For immunoprecipitation (IP), cells were lysed with IP buffer [50 mM Tris–HCl (pH 7.5), 150 mM NaCl, 1 mM EDTA, 1% NP-40] supplemented with a protease inhibitor cocktail for 15 min on ice. Whole cell lysates were centrifuged at 12 000 × *g* for 10 min. The supernatants were incubated with anti-FLAG beads or anti-PRRX1, anti-Ku70, anti-Ku80, and anti-DNA-PKcs antibodies, along with Protein A/G magnetic beads (Bimake, B23202), overnight at 4°C. After incubation, the beads were washed three times with IP buffer. The bound proteins were eluted with 2× SDS loading buffer, heated at 95°C for 10 min, loaded onto an SDS–PAGE gel, and immunoblotted with the indicated antibodies.

For G1/S synchronization, U2OS cells were treated with 2.5 μM thymidine for 16 h, washed three times with PBS, and cultured with fresh medium for 8 h. Cells were then treated with 2.5 μM thymidine for another 16 h to allow cells to be blocked in the G1/S phase. For M phase synchronization, U2OS cells were treated with 0.1 μg/ml colchicine (Beyotime, ST-1173) for 12 h.

### Sucrose density gradient centrifugation

To isolate and confirm the endogenous protein complexes associated with PRRX1, sucrose density gradient centrifugation (SDGC) assay was performed following published protocol [35]. A linear gradient was prepared using 10% and 40% (w/w) sucrose solutions with the BioComp Gradient Master (RRID: SCR_025583). Cells were lysed with SDGC lysis buffer [20 mM Tris–HCl (pH 7.5), 150 mM NaCl, 1 mM EDTA, 250 mM sucrose, 0.5% NP-40) supplemented with a protease inhibitor cocktail for 15 min on ice. After centrifugation at 12 000 × *g* for 5 min at 4°C, the supernatants (input) were loaded onto the sucrose gradient. Ultracentrifugation was performed using an SW40T rotor (Beckman; RRID: SCR_025331) at 100 000 × *g* for 16 h at 4°C. Aliquots from each fraction were analyzed by SDS–PAGE and IB to visualize the components.

### Chromatin fractions extraction

Following a previously described method with slight modifications [36], cytoplasmic soluble fractions were obtained using ice-cold lysis buffer [10 mM HEPES–NaOH (pH 8.0), 10 mM KCl, 1.5 mM MgCl_2_, 0.5 mM β-mercaptoethanol] supplemented with a protease inhibitor cocktail. Cells were lysed on ice for 15 min, followed by the addition of 0.2% NP-40 for 2 min. The lysates were centrifuged at 16 000 × *g* for 10 min at 4°C, and the pellets were washed at least twice with cold PBS. The washed pellets were resuspended in cold nuclei lysis buffer [10 mM Tris–HCl (pH 7.5), 300 mM NaCl, 2 mM MgCl_2_, 0.5% NP-40, 1 mM DTT] and incubated for 10 min to extract soluble nuclear proteins. After centrifugation at 16 000 × *g* for 10 min at 4°C, the remaining pellets were treated with 0.25 M HCl and incubated overnight at 4°C to extract histone and chromatin-associated proteins. The following day, the samples were centrifuged at 16 000 × *g* for 10–15 min, and the supernatants were collected as chromatin fractions. To neutralize the HCl, 2.5 M NaOH was added to the chromatin fractions before separation by SDS–PAGE.

### Expression and purification of recombinant proteins

Prokaryotic expression constructs of PRRX1 (pMAL-c2X and pET-28a) and Ku70/Ku80 (pGEX-4T-1) were transformed into the BL21 (DE3) *Escherichia coli* strain. When bacterial cultures reached an OD_600_ of 0.6–0.8, protein expression was induced with 0.2 mM Isopropyl β-D-thiogalactoside (IPTG) at 25°C for 12 h (PRRX1) or 16°C for 18 h (Ku70/Ku80). Bacteria were lysed by sonication in lysis buffer [20 mM Tris–HCl (pH 8.0), 150 mM NaCl, 0.5 mM EDTA, 0.1% Triton X-100], and lysates were cleared by centrifugation at 4°C. Supernatants were incubated with Ni-IDA 6FF resin (20 mM imidazole), amylose resin, or glutathione agarose beads, depending on the tag. Beads were washed with 10 column volumes of lysis buffer, and proteins were eluted with buffer containing either 200 mM imidazole, 10 mM maltose, or 20 mM reduced glutathione. MBP-PRRX1 proteins were further purified using molecular sieve chromatography and dialyzed in buffer [20 mM Tris–HCl (pH 7.5), 200 mM NaCl, 10% glycerol]. Proteins were concentrated using Amicon Ultra-4 filters, aliquoted, and stored at −80°C. Concentrations were estimated using SDS–PAGE stained with Coomassie blue.

### *In vitro* pull-down assay

For the MBP pull-down assay, 2.5 μg of MBP or MBP-PRRX1 (PA/PB) was incubated with ∼3 μg of GST or GST-Ku70/Ku80, 1 μg of His-Ku70/Ku80 heterodimer (Sino Biological, CT018-H07B), or 2 μg of His-PRRX1 (PA/PB) in 400 μl of NETN buffer [20 mM Tris–HCl (pH 8.0), 100 mM NaCl, 0.5 mM EDTA, 0.5% NP-40] per sample at 4°C for 2 h. Amylose resin (20 μl) was added to capture the protein complex, followed by constant shaking for 2 h. The supernatant was removed, and the resin was washed at least three times with 800 μl of the same buffer. Bound proteins were eluted with 40 μl of 2× SDS loading buffer, and eluates were analyzed by western blotting using the indicated antibodies.

For the DNA pull-down assay, pre-reactions were conducted in 200 μl of NETN buffer containing 0.1 μg/μl BSA, 20 pmol of biotinylated DNA substrate, and 25 μl of streptavidin agarose (Invitrogen, S951) at RT for 1 h. Subsequently, ∼100 fmol of purified MBP or various MBP-tagged PRRX1 proteins were added, and samples were incubated at 4°C for 2 h. Beads were washed three times with 500 μl of buffer each time and boiled in SDS loading buffer at 95°C for 5 min for western blot analysis.

### Chromatin immunoprecipitation

EJ5 U2OS cells were transfected with the indicated plasmids for 24 h or infected with lentiviruses expressing shRNAs for 48 h, followed by transfection with pCMV-I-SceI. After 24 h of incubation, cells were cross-linked with 1% formaldehyde and lysed in SDS-containing lysis buffer. The lysates were sonicated and subjected to IP using the indicated antibodies. Immunoprecipitated complexes were captured with protein A/G magnetic beads, and cross-linking was reversed by incubation at 65°C. Proteins were digested with proteinase K, and the DNA was purified. To analyze the damaged regions (the DSB site and a control site), the purified DNA was amplified using the indicated primers listed in Supplementary Table S3.

### Electrophoretic mobility shift assay

The indicated concentrations of MBP-PRRX1A (MBP-PA), with or without dsDNA, were combined with 200 nM of unlabeled dsDNA (blunt, 54 bp) substrate in 20 μl of binding buffer containing 20 mM Tris–HCl (pH 7.4), 10 mM MgCl_2_, 2.5% glycerol, 0.05% NP-40, and 0.1 μg/μl BSA. After a 15-min incubation at RT, the reactions were terminated by adding 4 μl of 6× DNA loading buffer (NEB), resolved on a 3% agarose gel stained with ethidium bromide, and analyzed using the Bio-Rad MP imaging instrument (RRID: SCR_019037).

### MST assay

Purified His-PA in optimized MST buffer [20 mM Tris–HCl (pH 7.4), 150 mM NaCl, 10 mM MgCl_2_, 2.5% glycerol, 0.05% Tween 20] was mixed with 6-FAM-labeled dsDNA as previously described. After a 10-min incubation at RT, the reaction mixtures were enclosed in premium-coated glass capillaries and loaded into the Monolith NT.115 instrument (NanoTemper). Measurement procedures and Kd value analysis were conducted using the corresponding software MO.Control (v1.4.2).

### DNA–PK kinase assay

DNA–PK activity was assessed using a DNA-PK Kinase Kit (Promega, V4106) following the manufacturer’s protocol and incorporating slight modifications based on prior studies [16, 26]. The kinase reaction system included DNA–PK kinase enzyme, reaction buffer, activation buffer containing 100 μg/ml Calf thymus (CT) DNA, a mixture of γ-^32^P-ATP (10 mCi/ml; PerkinElmer, NEG502A100UC) and cold ATP (Solarbio, A9130), and recombinant C-MYC protein (HCUSABIO, CSB-EP015270HU) as the substrate. Each reaction contained the DNA–PK holoenzyme in a solution comprising 1 μl ATP mixture (with 2 μCi γ-^32^P-ATP), 10 ng/μl CT-DNA, 1.5 μg C-MYC, 100 U DNA–PK kinase (∼0.8 μl), and different purified MBP-tagged PRRX1 proteins (∼0.2 μM). The reaction mixture was incubated at 30°C for 15 min, diluted with 5× SDS loading buffer, boiled at 95°C for 5 min, and analyzed on an SDS–PAGE gel. DNA–PK activity was quantified as the band signal of ^32^P-labeled C-MYC on the dried gel using the GE Typhoon FLA9500 phosphoimager (RRID: SCR_019946).

### NHEJ and HR reporter assays

NHEJ and HR reporter assays were conducted following previously established protocols [37]. U2OS cells were treated with 2 μM NU7441 or 5 μM Olaparib for 6 h. Subsequently, 2 μg of linearized reporter plasmids, either pimEJ5GFP (miaolingbio, P31613; RRID: Addgene_44026) or pDRGFP (a gift from Dr Pingkun Zhou; RRID: Addgene_26475), digested with I-SceI meganuclease (NEB, R0694S), were co-transfected with 0.4 μg of either an EV (expressing mCherry) or mCherry-PRRX1 constructs into cells plated in six-well plates. After 24 h, cells were harvested, and GFP-positive cells were quantified using the BD LSRFortessa X-20 Cell Analyzer (RRID: SCR_025285). The relative repair efficiency was calculated as the proportion of GFP/mCherry double-positive cells to total mCherry-positive cells. Data represent the means of at least three independent experiments.

### Cell survival assay

Hs746T cells were infected with shPRRX1 lentivirus for 48 h, trypsinized, and seeded at density of 3 × 10^3^/well in 96-well plates and exposed to IR. MEFs were seeded in 96-well plates at density of 10^3^/well for 24 h and exposed to increasing doses of ETP, CPT, or IR. After 96 h, cell survival was performed using the CCK-8 Kit (Beyotime, C0038) and Molecular Devices SpectraMax iD3 microplate reader (RRID: SCR_023920). Cells without treatment were used as controls. The survival fraction was calculated as OD450 [(sample blank)/(control blank)]. Results are presented as the mean ± SEM from at least three independent experiments.

### Metaphase spread

MEFs were incubated with 0.1 μg/ml colchicine at 37°C for 4 h, then collected and swollen in prewarmed 75 mM KCl at 37°C for 20 min. After centrifugation, the cells were fixed with Carnoy’s buffer (3:1 methanol:acetic acid) at RT for 10 min. The cells were centrifuged for 4 min at 1000 × *g* and the supernatant was aspirated. The cells were resuspended in Carnoy’s buffer twice. The cell suspension was dropped onto slides and dried for at least 15 min. Slides were stained with Wright Giemsa solution (20×; Beyotime, C0131), and genomic instability was assessed by counting cells with chromosomal breaks.

### RNA isolation and reverse transcription quantitative PCR (RT-qPCR)

Total RNA from MEFs or U2OS cells was isolated using RNAiso Plus (TAKARA, 9109) according to the manufacturer’s instructions. One microgram of total RNA was reverse transcribed into cDNA using HiScript II Q RT SuperMix (Vazyme, R222-01). The resulting cDNA was then analyzed by quantitative PCR (qPCR) using SYBR Green qPCR Mix (Monad, MQ00801S). The amplification protocol consisted of an initial denaturation at 95°C for 5 min, followed by 40 cycles of 95°C for 15 s, and 60°C for 20 s. Gene expression levels were normalized to the Ct values of *Gapdh* or *ACTB* (control) using the 2^−ΔΔCt^ method. Reactions were conducted on a Bio-Rad CFX Connect™ Real-Time PCR Detection System (RRID:SCR_018064). Primer sequences for the qPCR are listed in Supplementary Table S3. RNA sequencing of MEFs was performed and analyzed by Novogene (Beijing, China).

### wGII, genomic scar signature, and survival analyses

Allele-specific segmented copy number data, clinical survival data, and PRRX1 RNA expression data (log-transformed transcripts per million) from the TCGA STAD cohort were downloaded using the R package TCGAbiolinks (RRID: SCR_017683). The samples were categorized based on the median *PRRX1* expression level. The weighted genomic integrity index (wGII) for all samples was recalculated following previous studies [38, 39]. Genomic scars [loss of heterozygosity (LOH), telomeric allelic imbalance (TAI), and large-scale transition (LST)] were calculated using the R package scarHRD (v0.1.1), based on relevant studies [40, 41]. Statistical significance was assessed using the two-sided Wilcoxon rank-sum test (Mann–Whitney *U*-test). Overall survival analysis for gastric cancer cases (*n* = 531) was conducted using the best cutoff method on Kaplan–Meier Plotter (https://kmplot.com/analysis/; RRID: SCR_018753). For samples with radiotherapy information (*n* = 78), grouping was performed based on *PRRX*1 expression levels. Survival differences among groups were assessed using TCGAanalyze_survival, and visualizations were generated using R v4.2 (RRID: SCR_001905).

### Protein structure prediction and docking

The predicted structural model of human PRRX1 (model: AF G3V2N3 F1) was downloaded from the AlphaFold Protein Structure Database (https://alphafold.ebi.ac.uk; RRID: SCR_023662) [42], and docked with the structures of dsDNA (PDB: 5J3G) or the SAP domain of human Ku70 (PDB: 1JJR) from the RCSB PDB (RRID: SCR_008227) as ligands using the ClusPro 2.0 webserver (https://cluspro.bu.edu/login.php; RRID: SCR_018248) [43]. The docking results were analyzed and prepared for figures in UCSF ChimeraX-1.7 (RRID: SCR_015872) [44].

### Statistics and reproducibility

All statistical analyses were performed using GraphPad Prism (v.10.0) (RRID: SCR_002798) or Microsoft Excel 2019 (RRID: SCR_016137). Statistical significance was determined using two-tailed Student’s *t*-test or ANOVA, as appropriate. A *P*-value of <.05 was considered significant. The number of cells or replicates (*n*) for each experiment is indicated in the figures or legends. Data for bar and line graphs are presented as mean ± SEM unless otherwise specified in the legends. Immunofluorescence micrographs are representative of at least two independent experiments using the indicated cells with the same treatment. Western blot data were obtained from the respective experiment, processed in parallel, and are representative of three independent experiments unless otherwise noted in the legends.

## Results

### PRRX1 interacts with Ku70 *in vitro* and *in vivo*

**Figure 1.**
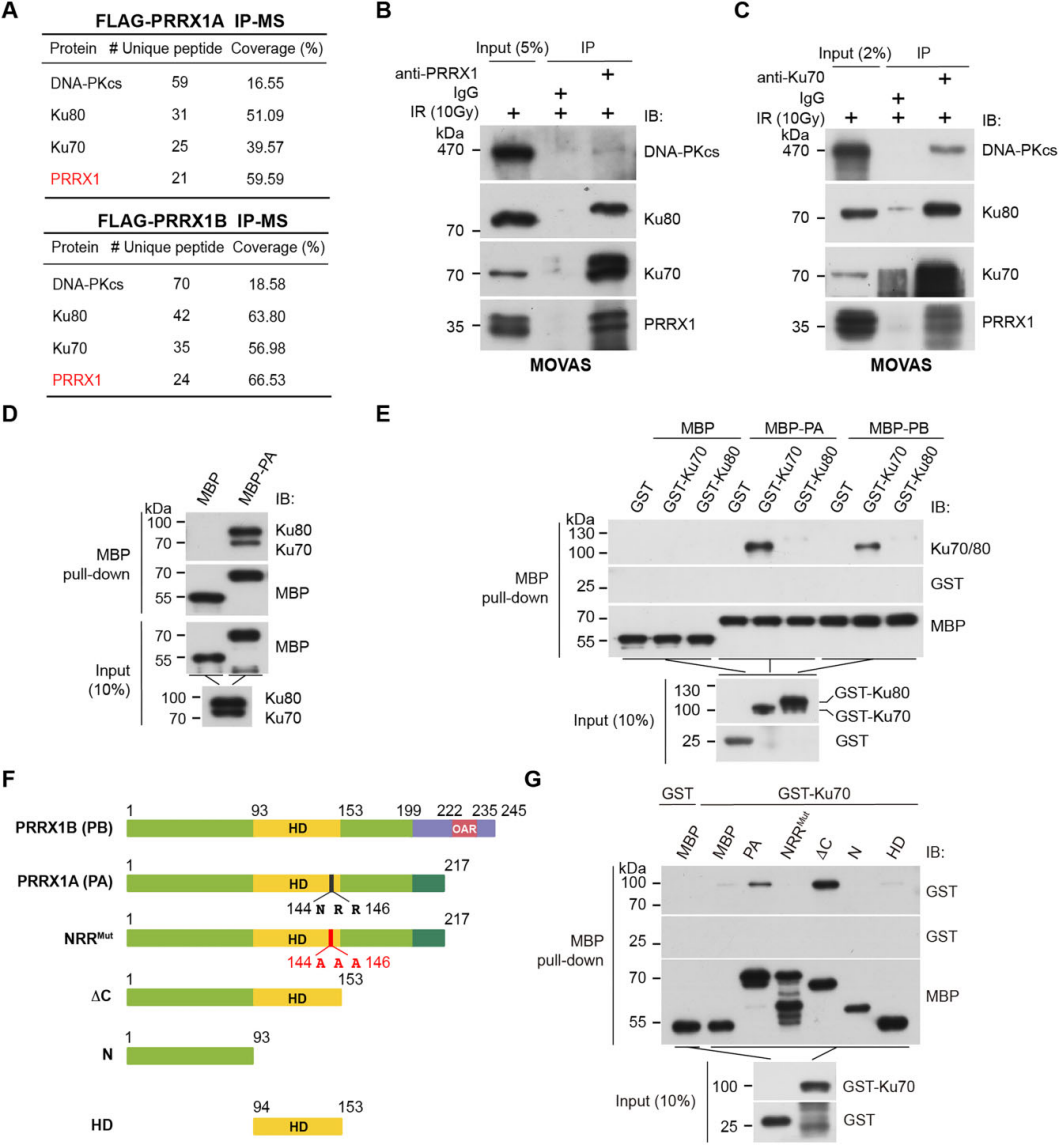
PRRX1 interacts with Ku70. (**A**) Proteins identified by IP of FLAG-PRRX1 followed by MS in glioblastoma cells. Components of the NHEJ core complex and PRRX1 are listed. Lysates of irradiated MOVAS cell were precipitated with anti-PRRX1 (**B**) or anti-Ku70 (**C**) antibodies followed by IB with indicated antibodies. MBP pull-down assays using recombinant MBP-PA to co-purify His-tagged Ku70/80 heterodimer (**D**) and GST-tagged Ku70 or Ku80 (**E**). Co-purified products were immunoblotted with indicated antibodies. (**F**) The diagram depicting the long (PB) and short (PA) isoforms of PRRX1 along with PA’s mutants and truncations. Numbers indicate amino acid residues. OAR: the otp/aristaless/rax domain. (**G**) MBP pull-down assays using MBP-PA truncations to co-purify GST-Ku70. Co-purified products were immunoblotted with indicated antibodies. All experiments were performed for at least three times and representative blots were shown.

The HD transcription factor PRRX1 plays a pivotal role in mesodermal development and is implicated in various cancers. In glioblastoma, PRRX1 promotes propagation of GICs, as well as tumor invasion and angiogenesis [45, 46]. To gain a comprehensive understanding of PRRX1’s functions, we sought to identify its associated proteins. We performed affinity purification of PRRX1 using GICs stably expressing Flag epitope-tagged PRRX1 isoforms - PA or PB - and carried out affinity purification and mass spectrometry (AP-MS) analysis. Unexpectedly, we found that several NHEJ factors, including Ku70 (encoded by *XRCC6*), Ku80 (encoded by *XRCC5*), and DNA-PKcs (encoded by *PRKDC*), are among the top hits (Fig. 1A and Supplementary Table S1). Of note, none of the HR components were identified through AP-MS. We then repeated the AP-MS analysis in untreated or DNA-damaged U2OS cells overexpressing Flag-tagged PA. Consistently, several DDR factors, particularly those involved in the NHEJ, as well as chromatin associated proteins, were identified as PRRX1-associated proteins (Supplementary Fig. S1A and B and Supplementary Table S1). To validate the AP-MS data, we first performed co-immunoprecipitation (co-IP) experiments in HEK293T cells transiently transfected with plasmids expressing Flag-tagged PA/PB. As shown in Supplementary Fig. S2A–C, PRRX1 interacted with all three components of the DNA–PK complex, Ku70, Ku80, and DNA-PKcs. Previous studies have indicated that the association between DNA-PKcs and the Ku70/Ku80 heterodimer is DNA dependent [47]. However, we revealed that the addition of DNase I did not disrupt the interaction between PRRX1 and the DNA–PK complex (Supplementary Fig. S2B), suggesting that this interaction is DNA-independent. We next carried out reciprocal co-IP assay in MOVAS cells and verified the endogenous interactions among PRRX1, Ku70/80, and DNA-PKcs (Fig. 1B and C and Supplementary Fig. S2D). Notably, the the association between PRRX1 and the DNA–PK complex is cell cycle independent (Supplementary Fig. S2E). Moreover, sucrose gradient fractions of U2OS and MEF cell extracts showed that a substantial portion of Ku70/80 and DNA-PKcs sedimented with PRRX1 (Supplementary Fig. S2F and G). Thus, we identified PRRX1 as a novel interacting partner of the DNA–PK complex, suggesting a potential role for PRRX1 in NHEJ.

To explore whether PRRX1 directly binds to Ku70/80 or DNA-PKcs, we examined the interactions between purified MBP-PRRX1 (Supplementary Fig. S3A) and recombinant Ku70 and Ku80 proteins (Supplementary Fig. S3B), or Ku70/80 and DNA-PKcs in lysates derived from the WT or Ku80 KO Chinese hamster ovary (CHO) cells (Supplementary Fig. S3C). The results showed that PRRX1 specifically interacts with Ku70 but not with Ku80 or DNA-PKcs (Fig. 1D and E and Supplementary Fig. S3C).

### The PRRX1 NRR motif and Ku70 SAP domain are crucial for mediating the PRRX1–Ku70 interaction

We next identified the specific region of PRRX1 required for its interaction with Ku70. A serials of MBP-fused PRRX1 truncations and mutations were generated, including the C-terminus-deleted PA (ΔC), the N-terminal region (N), the HD, and a mutant with conserved residues Asn144/Arg145/Arg146 altered (NRR^Mut^) (Fig. 1F). Pull-down assays revealed that the Ku70-binding capacity of PRRX1 depends on its HD and N-terminal region. Specifically, only the full-length and the C-terminus-deleted (ΔC) PA could bind to Ku70, while the NRR^Mut^ mutant failed to do so (Fig. 1G). Thus, the conserved NRR motif within the HD domain of PRRX1 is crucial to mediate its interaction with Ku70. Interestingly, the ΔC exhibited a higher affinity to GST-Ku70 than the full-length PRRX1, implying that the C-terminal region might limit its ability to bind Ku70. Moreover, the docking between PRRX1 and Ku70/80 showed that Asn144/Arg146 constitutes the interaction interface with the SAP domain of Ku70 (Supplementary Fig. S3D). Concordantly, MBP pull-down assays revealed that neither PA^N144A^ nor PA^R146A^ could associate with Ku70/80 (Supplementary Fig. S3E). To determine the PRRX1-interacting domain of Ku70, we generated a series of GST-tagged Ku70 truncations and performed pull-down assays (Supplementary Fig. S3F). Notably, our investigation revealed that among various segments of Ku70, only the C-terminal SAP domain possesses the capability to bind to PRRX1 (Supplementary Fig. S3G). Collectively, PRRX1 interacts with Ku70 both *in vitro* and *in vivo*, with the interaction likely mediated by the NRR motif of PRRX1 and the SAP domain of Ku70.

### PRRX1 rapidly translocates onto DNA damage sites

**Figure 2.**
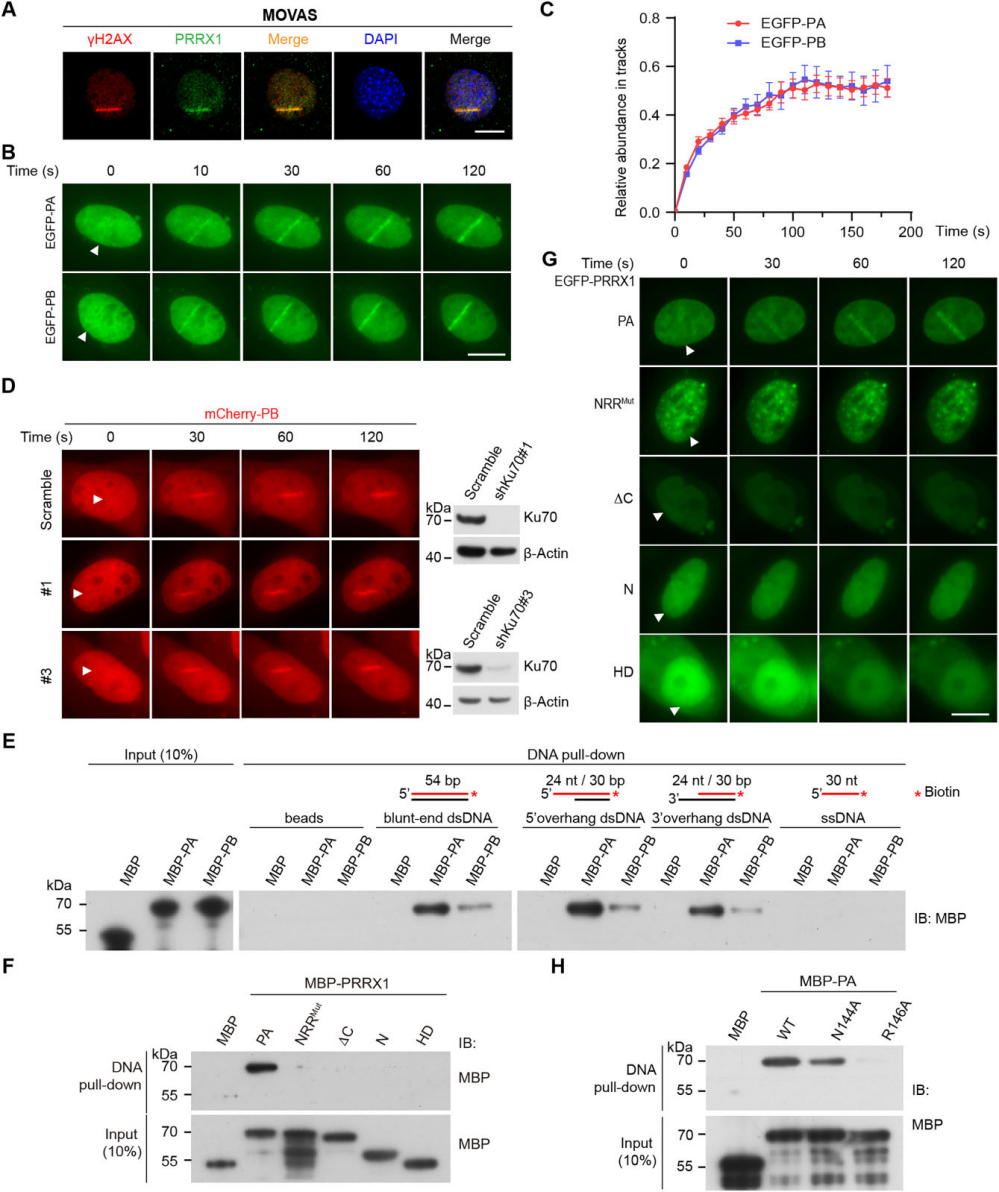
PRRX1 accumulates at DNA damage sites and directly binds to dsDNA. (**A**) MOVAS cells were subjected to LMI. Thirty minutes later, cells were fixed and subjected to immunofluorescence of anti-γH2AX and anti-PRRX1 antibodies. (**B**) U2OS cells were transfected with vectors expressing EGFP-PA/PB and subject to LMI. Fluorescence images were collected at indicated timepoints. (**C**) Quantification of panel (B). Data are presented as the mean fluorescence intensity change of irradiated stripes over background per cell (mean ± SEM). PA, *n* = 20 cells; PB, *n* = 20 cells. (**D**) Left panel: U2OS cells were transfected with vectors expressing mCherry-PB and shRNAs against Ku70 and subject to LMI. Fluorescence images were collected at indicated timepoints. Right panel: Cell lysates at left were collected for IB of indicated antibodies. (**E**) DNA pull-downs of various forms of biotinylated (red asterisks) ssDNAs and dsDNAs with MBP, MBP-PA, and MBP-PB. Samples were immunoblotted with an anti-MBP antibody. (**F**) DNA pull-downs of blunt-end biotinylated dsDNA with various MBP-tagged PRRX1 mutants and truncations. (**G**) U2OS cells were transfected with vectors expressing various forms of EGFP-tagged PRRX1 mutants and truncations and were subjected to LMI. Fluorescence images were collected at indicated timepoints. (**H**) DNA pull-downs of biotinylated dsDNA with MBP and MBP-PA mutants. Samples were immunoblotted with an anti-MBP antibody. All experiments were performed for at least three times and representative images/blots were shown. The triangles indicate irradiated tracks. Scale bars, 10 μm. s, seconds.

Next, we investigated whether PRRX1 could be recruited to DNA lesions. LMI followed by immunofluorescence in MOVAS cells revealed that endogenous PRRX1 accumulated at damaged tracks marked by γH2AX, an indicator of the DNA damage (Fig. 2A). We conducted live cell imaging on U2OS cells expressing EGFP-PA/PB to delineate the dynamics of PRRX1 recruitment to DNA damage sites. The results demonstrated that the recruitment of PRRX1 to DNA damage sites is rapid, reaching its peak at 2 min after LMI (Fig. 2B and C). We next studied the impact of various DSB factors, including PARP1, 53BP1, Ku70, DNA-PKcs, ATM, and the MRN complex [48–51], on PRRX1 accumulation at DNA damage sites. These proteins can initiate downstream signaling cascades leading to the activation of NHEJ repair pathways. However, our findings indicated that shRNA-mediated knockdown of Ku70 (Fig. 2D), inhibition of PARP1, DNA–PK, ATM, or the MRN complex (Supplementary Fig. S4A), or deletion of 53BP1 (Supplementary Fig. S4B), did not affect the recruitment of PRRX1 to DNA damage sites. These findings indicate that PRRX1 recruitment at DNA damage sites is independent of PARP1, Ku70, DNA–PK, or ATM, suggesting that it acts in the very early stage of DNA damage recognition and response. Notably, the recruitment kinetics of EGFP tagged PRRX1 to DNA damage sites were almost identical to those of EGFP-Ku70 (Supplementary Fig. S4C).

### PRRX1 binds to DNA in an NRR motif-dependent manner

We then performed the biotinylated DNA pull-down using various forms of oligonucleotides with random sequences. MBP-PRRX1 exhibited a strong affinity to dsDNA with blunt ends, 3′ or 5′ overhangs, but showed no binding to ssDNA (Fig. 2E). It is worth noting that the shorter isoform, PA, demonstrated stronger DNA-binding affinity compared with the longer isoform, PB. Electrophoretic mobility shift assay and the MST assay demonstrated the MBP-PA or His-PA binds to both blunt-ended dsDNA and 5′ overhang dsDNA with various overhang lengths (Supplementary Fig. S5A and B). Importantly, only the full-length PA (MBP-PA) was captured by dsDNA. The NRR motif of PA, which is essential to mediate the interaction with Ku70, is also required for PRRX1’s binding to DNA, as the NRR^Mut^ mutation completely abolished this capacity (Fig. 2F). In addition, PRRX1 truncation proteins lacking the C-terminus (ΔC) or retaining only the N-terminus (N) or the HD failed to bind DNA (Fig. 2F). Thus, full-length PRRX1 is required for DNA binding *via* the NRR motif. Consistently, LMI experiments showed that only the full-length EGFP-PA, but not its truncations or mutations, could be recruited to DNA lesions (Fig. 2G). These findings indicate that the NRR motif within the HD of PRRX1 is required for its DNA binding and accumulation at DNA damage sites.

Referring to the electrostatic potential map of human PRRX1 obtained from the AlphaFold Protein Structure Database [42], it is observed that the HD region possesses a negative electrostatic potential and interacts with the DNA major groove (Supplementary Fig. S5C). Additionally, ClusPro docking simulation [43] revealed that the conserved residues Asn144/Arg145/Arg146, located at the helix α3 of HD, could bind to bases (by Asn144) and DNA backbone (by Arg145/Arg146) through hydrogen bonds, nonbonded electrostatic and hydrophobic interactions (Supplementary Fig. S5D and E). Indeed, the DNA pull-down assay showed that PA^N144A^ and PA^R146A^ mutations reduced or abolished the interaction with dsDNA, respectively (Fig. 2H). Therefore, PRRX1 likely localizes to DNA breaks through directly binding to DNA. Interestingly, our results indicate that the NRR motif within the HD is essential for PRRX1’s interaction with both Ku70 and DNA.

### PRRX1 facilitates the recruitment and assembly of the DNA–PK complex on the chromatin

**Figure 3.**
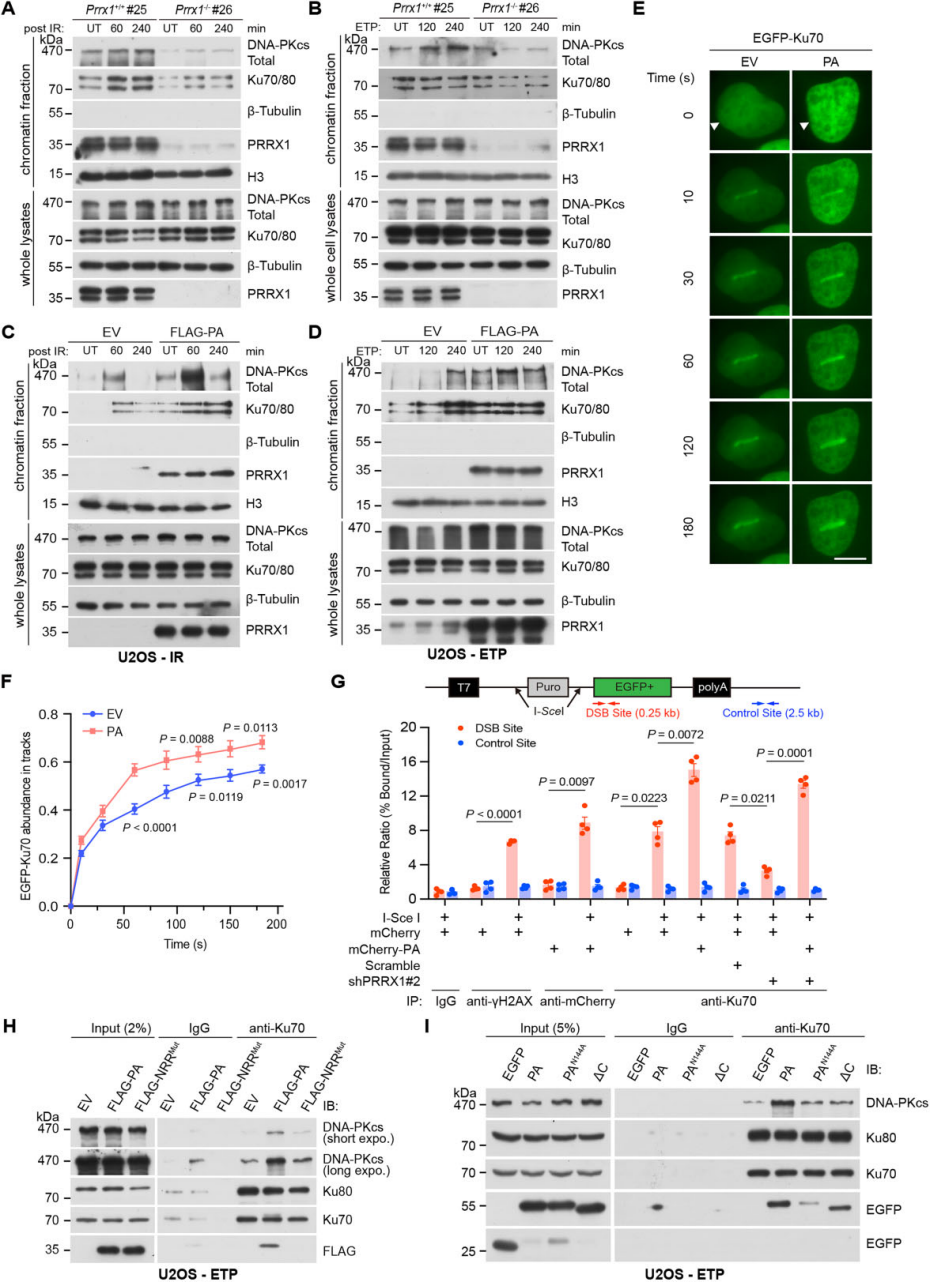
PRRX1 facilitates the recruitment of Ku70/80 and the assembly of DNA–PK on chromatin. WT and *Prrx1*^−/−^ MEF cells were treated with 5 Gy irradiation (**A**) or 10 μM ETP (**B**) at indicated time. Chromatin fractions (top panel) and whole cell lysates (bottom panel) were immunoblotted with indicated antibodies. U2OS cells were transfected with EV or FLAG-PA for 36 h. Chromatin fractions (top panel) and whole cell lysates (bottom panel) were extracted at indicated time post 5 Gy irradiation (**C**) or 10 μM ETP (**D**) and subjected for IB with indicated antibodies. U2OS cells were co-transfected with vectors expressing EGFP-Ku70 and pHAGE-PA. After transfection, cells were subjected to LMI (**E**). Scale bar, 10 μm. The relative fluorescent intensities of EGFP-Ku70 in cells of panel (E) were quantified. Data are mean ± SEM; 15 cells per each group were analyzed from two independent experiments (**F**). Statistical analysis was performed using two-tailed unpaired *t*-tests. (**G**) Top panel: Diagram illustrating sites of DSBs and chromatin enrichment analyisis in EJ5 U2OS cells. Bottom panel: Cells were treated as indicated, followed by ChIP using indicated antibodies and subsequent qPCR. U2OS cells were transfected with EV, FLAG-PA, and FLAG-NRR^Mut^ (**H**) and EGFP-PA^N144A^ and EGFP-ΔC (**I**). Cell lysates were co-immunoprecipitated with anti-Ku70 antibody followed by IB with indicated antibodies. UT, untreated.

Since PRRX1 can bind to both DNA and Ku70, we investigated whether PRRX1 affects Ku dynamics at damage sites and subsequent loading of DNA-PCcs. To this end, we examined DSB-induced recruitment of endogenous Ku70/80 and DNA-PKcs to chromatin in MEFs. MEFs were derived from *Prrx1*-KO and WT embryos (Supplementary Fig. S6A and B). Chromatin fractionation analysis revealed that the enrichment of Ku70/80 and DNA-PKcs on chromatin was markedly reduced in *Prrx1*^−/−^ cells compared with WT cells following ETP or IR treatment (Fig. 3A and B and Supplementary Fig. S6C and D). Conversely, in PRRX1-overexpressing U2OS cells, the chromatin enrichment of Ku70/80 and DNA-PKcs was markedly increased (Fig. 3C and D). LMI assays further showed that PRRX1 overexpression enhanced the recruitment of EGFP-Ku70 to DNA lesions (Fig. 3E and F). To further investigate the role of PRRX1 in Ku70 recruitment to DSB sites, we conducted chromatin immunoprecipitation (ChIP) analysis in EJ5 U2OS cells, where site-specific DSBs were induced by I-SceI digestion. ChIP assays were performed using antibodies against IgG, γH2AX, mCherry (for mCherry or mCherry-PA), or Ku70. The immunoprecipitated DNA was analyzed by qPCR using primers targeting DSB and control sites, as illustrated in Fig. 3G. PRRX1 knockdown significantly decreased the enrichment of DNA fragments in Ku70 immunoprecipitants. In contrast, PRRX1 overexpression enhanced the enrichment of DNA fragments in Ku70 immunoprecipitants. Moreover, PRRX1 overexpression reversed the reduced enrichment of DNA fragments by anti-Ku70 antibodies. Taken together, PRRX1 promotes the loading of Ku70/80 and DNA-PKcs at DSB lesions.

Next, we asked whether PRRX1 could facilitate the assembly of the DNA–PK complex. First, DNA pull-down assays demonstrated that blunt-end biotinylated dsDNA enriched more Ku70/80 and DNA-PKcs from lysates of PRRX1-overexpressing cells compared with control cells (Supplementary Fig. S6G). Moreover, we noted that the presence of PRRX1 enhanced the association between Ku70/80 and DNA-PKcs, which was abolished if the Ku70- and DNA-binding NRR motif was mutated (Fig. 3H and Supplementary Fig. S6E and F). Remarkably, both PRRX1^N144A^ (retaining DNA- but not Ku70 binding) and ΔC (retaining Ku70- but not DNA binding) lost their abilities to facilitate the assembly of DNA–PK (Fig. 3I). Therefore, both Ku- and DNA-binding capacities of PRRX1 are required for stimulating the assembly of the DNA–PK complex on chromatin.

### PRRX1 stimulates the kinase activity of DNA–PK *in vitro* and *in vivo*

**Figure 4.**
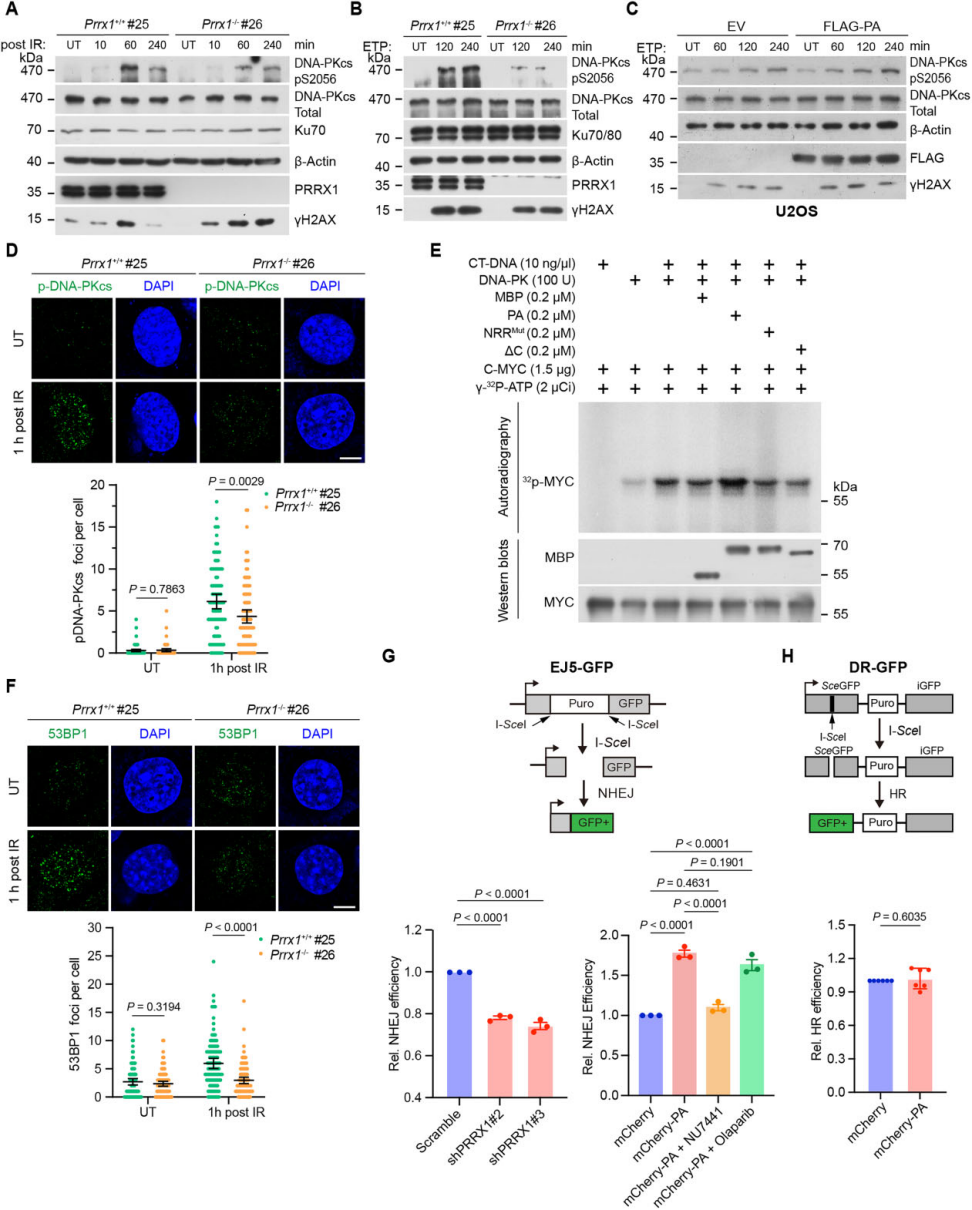
PRRX1 activates DNA–PK to promote NHEJ. WT and *Prrx1*^−/−^ MEF cells were treated with 5 Gy irradiation (**A**) or 10 μM ETP (**B**) and harvested at indicated times. Cell lysates were immunoblotted with indicated antibodies. (**C**) U2OS cells were transfected with EV or FLAG-PA for 36 h followed by treating with 10 μM ETP. Cell lysates were immunoblotted with indicated antibodies. WT and *Prrx1*^−/−^ MEF cells were treated with 5 Gy irradiation for 1 h followed by immunofluorescence (top panel) and quantification (bottom panel) of phosphorylation at Ser2056 of DNA–PK (**D**) or 53BP1 (**F**) foci after irradiation. Statistical analysis was performed using two-tailed unpaired *t*-tests. Each point represents one cell; 100 cells quantified in each group were obtained from two independent experiments. Scale bars, 10 μm. (**E**) In the DNA–PK kinase assay, indicated components were mixed and the C-MYC protein was used as the substrate. Autoradiography and IB using indicated antibodies were performed. (**G**) Top panel: The diagram showing the EJ5-GFP reporter assay assessing the efficiency of NHEJ. Bottom panel: Percentages of NHEJ (GFP^+^) cells were analyzed by flow cytometry and fold changes were normalized to cells transfected with empty mCherry-expressing vector. Data are mean ± SEM. Statistical analysis was performed using one-way ANOVA. *n* = 3 biologically replicates. (**H**) Top panel: The diagram showing the DR-GFP reporter assay assessing the efficiency of HR. Bottom panel: Percentages of HR (GFP^+^) cells were analyzed by flow cytometry and fold changes were normalized to cells transfected with empty mCherry-expressing vector. Data are mean ± SEM. Statistical analysis was performed using two-tailed unpaired *t*-test. *n* = 6 biologically replicates.

The DSB repair by NHEJ is initiated by the recognition and binding of the Ku70/80 heterodimer to broken DNA ends, which subsequently recruits DNA-PKcs and leads to its auto-phosphorylation at Ser2056 and Thr2609, a hallmark of NHEJ activation [52, 53]. PRRX1, along with the cofactor FOXM1, was reported to activate the transcription of *PRKDC*, the gene encoding DNA-PKcs, in pancreatic cancer cells [34]. However, *Prrx1*^−/−^ MEFs did not display significant alterations in messenger RNA (mRNA) levels of *Prkdc* and *Xrcc6* (Supplementary Fig. S7B and Supplementary Table S2), and the protein level of DNA-PKcs only slightly decreased. Importantly, the phosphorylation levels of DNA-PKcs at Ser2056 post IR or ETP was strikingly decreased in *Prrx1*^−/−^ MEFs (Fig. 4A and B and Supplementary Fig. S7A). As expected, levels of phosphorylated Ser2056 were modestly elevated in U2OS cells expressing Flag-tagged PA (Fig. 4C). Consistently, significantly fewer phosphorylated DNA-PKcs foci were detected in irradiated *Prrx1*^−/−^ MEFs than those in irradiated WT MEFs (Fig. 4D). Of note, the mRNA levels of *PRKDC* and *XRCC6* were not changed in PRRX1-overexpressing U2OS cells (Supplementary Fig. S7D). To test whether PRRX1 could directly stimulate the activity of DNA–PK holoenzyme (Ku70/80 included), we performed kinase assays using purified His-tagged MYC as the phosphorylation substrate. As anticipated, the presence of dsDNA activated DNA–PK. Critically, the full-length PA significantly augmented MYC phosphorylation, whereas PRRX1 with mutated NRR or truncated C-terminus did not exhibit this effect (Fig. 4E).

The number of 53BP1 foci, the indicator of the NHEJ repair, was significantly reduced in irradiated *Prrx1*^−/−^ MEFs, suggesting impaired NHEJ in the absence of PRRX1 (Fig. 4F). Next, we employed the EJ5-GFP and DR-GFP reporter assays to directly assess the influence of PRRX1 on DSB repair *via* NHEJ or HR [54]. As expected, knocking down the expression of PRRX1 compromised the NHEJ repair efficiency (Fig. 4G and Supplementary Fig. S7C). Remarkably, PRRX1 overexpression led to a significant increase in NHEJ repair effeciency but had no effect on HR repair. Of note, the enhancement of NHEJ by PRRX1 overexpression was abolished by DNA–PK inhibitor NU7441 but only slightly affected by PARP1 inhibitor Olaparib (Fig. 4G and H and Supplementary Fig. S7D). Thus, PRRX1 facilitates the activation of DNA–PK to promote NHEJ repair.

### The oligomerization of PRRX1 is essential for the NHEJ repair

**Figure 5.**
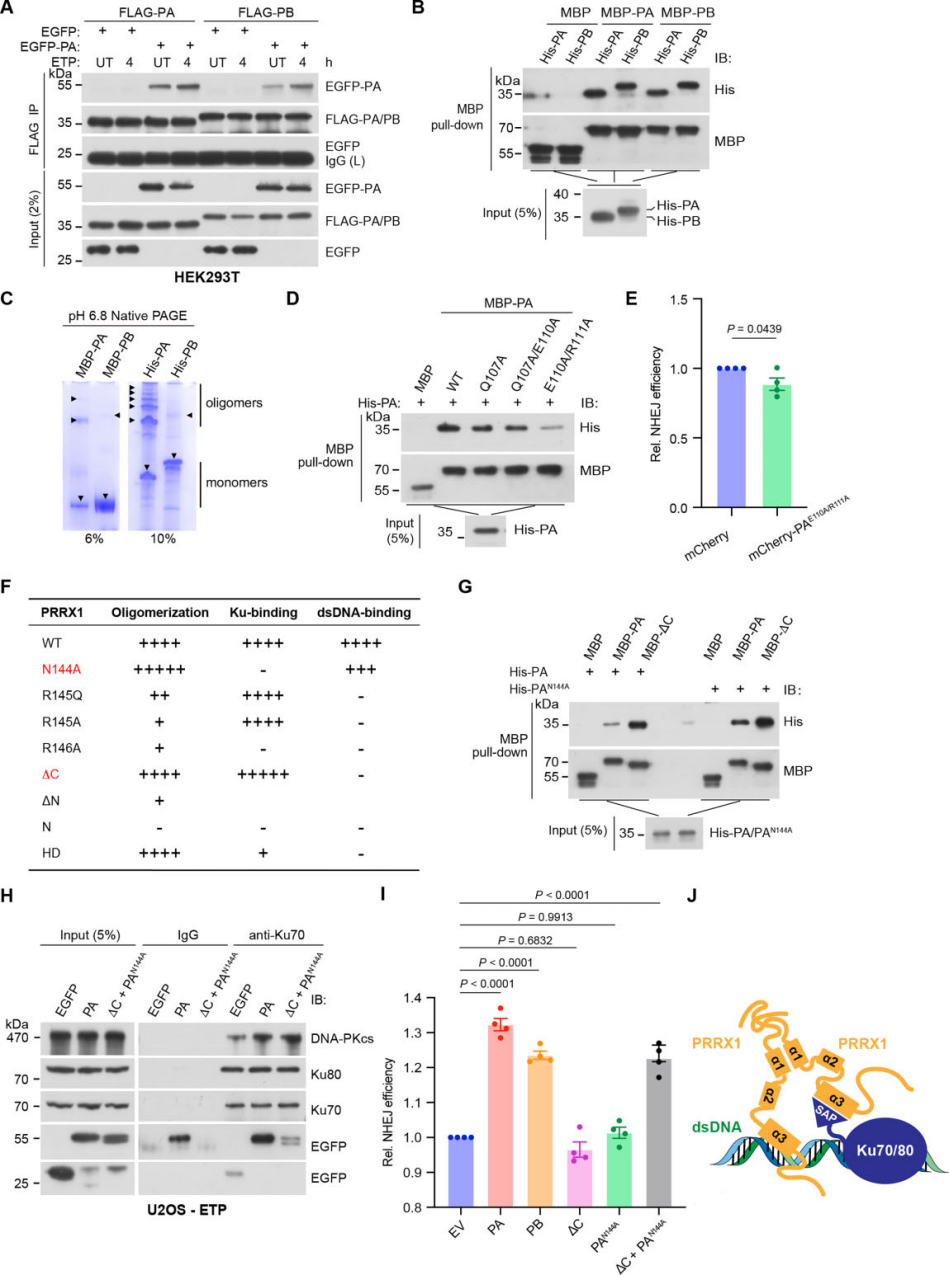
PRRX1 oligomerizes to mediate NHEJ repair. (**A**) HEK293T cells were co-expressed with EGFP-PA and FLAG-PA or FLAG-PB followed by 10 μM ETP treatment. Lysates were immunoprecipitated with anti-FLAG beads followed by IB with indicated antibodies. (**B**) MBP pull-down assays using recombinant MBP, MBP-PA, and MBP-PB to co-purify His-PA and His-PB. Co-purified products were immunoblotted with indicated antibodies. (**C**) Native PAGE of *in vitro* purified MBP-PA and MBP-PB (left panel) and His-PA and His-PB (right panel). The triangles indicate putative monomers and oligomers. (**D**) MBP pull-down assays using recombinant MBP, MBP-PA (WT), and indicated PA mutations to co-purify His-PA. Co-purified products were immunoblotted with indicated antibodies. (**E**) EJ5-GFP NHEJ reporter assays of U2OS cells overexpressing mCherry and mCherry-PA^E110A/R111A^. *n*= 4 biologically replicates. Statistical significance was determined by two-tailed unpaired *t*-test. (**F**) The table summarizing oligomer-forming, Ku- and DNA-binding capacities of various PRRX1 mutants and truncations. (**G**) MBP pull-down assays using recombinant MBP, MBP-PA, and MBP-ΔC to co-purify His-PA and His-PA^N144A^. Co-purified products were immunoblotted with indicated antibodies. (**H**) U2OS cells were transfected with vectors expressing EGFP, EGGP-PA, or EGFP-PA^N144A^ with EGFP-ΔC and subjected to ETP treatment. Cell lysates were immunoprecipitated with anti-Ku70 antibody followed by IB with indicated antibodies. (**I**) EJ5-GFP reporter assays of U2OS cells overexpressing indicated vectors (mCherry tagged). *n*= 4 biologically replicates. Statistical analysis was performed using one-way ANOVA. (**J**) The diagram depicting a model in which PRRX1 oligomerizes through its α1 helix to facilitate simultaneous interactions with dsDNA and Ku70/80.

As described earlier, PRRX1 binds to Ku70 or DNA *via* the same NRR motif in its HD domain. This prompt us to explore the possibility of PRRX1 oligomerization, which could enable simultaneous interaction with both Ku70 and DNA. We performed co-IP assays using extracts from HEK293T cells expressing Flag-PA/PB and EGFP-PA/PB, which revealed both homophilic and heterophilic interactions between PA and PB. Furthermore, we observed that these interactions were enhanced under IR and ETP conditions (Fig. 5A and Supplementary Fig. S8A). Meanwhile, MBP pull-down assays showed that purified MBP-PA/PB could co-purify His-tagged PA/PB (Fig. 5B). Furthermore, we observed that both PA and PB could form aggregates on native polyacrylamide gel electrophoresis (PAGE), with PA having a greater propensity for oligomer formation (Fig. 5C).

To investigate the regions of PRRX1 required for its oligomerization, we conducted MBP pull-down assays using a series of MBP-PA truncations to co-purify His-tagged PA/PB. We observed that the ΔN (9–217 aa) displayed reduced interaction with full-length PRRX1, whereas HD alone exhibited strong binding (Supplementary Fig. S8B). Interestingly, the N-terminal region alone could not bind to PRRX1 (Supplementary Fig. S8B), suggesting that both the N-terminal and the HD domain of PRRX1 are essential for the oligomeric formation. The molecular docking analysis predicted that several residues at the N-terminal helix α1 of the HD might be involved in the oligomerization (Supplementary Fig. S8C). Next, point mutants of these residues were employed to assess their association with WT His-PA. The results revealed that PRRX1^Q107A^ and PRRX1^Q107A/E110A^ slightly reduced the interaction, while PRRX1^E110A/R111A^ severely compromised the interaction (Fig. 5D). Consistently, PRRX1^E110A/R111A^ lost the ability to promote DSB repair by NHEJ (Fig. 5E). Properties of oligomerization, Ku- and dsDNA-binding in all mutants, and truncations were summarized in Fig. 5F.

We further assessed the oligomeric capability of PA with mutations at conserved residues 144–146 of the HD. The results indicated that PA^R145A^ and PA^R146A^ barely interacted with WT PA, while PRRX1^R145Q^, identified in cancerous patients, and PRRX1^N144A^ could bind to PA, with the latter exhibiting stronger association (Supplementary Fig. S8D). To examine the impact of PRRX1 oligomerization on NHEJ, we studied the combination of PA^N144A^ with ΔC, which respectively associate with dsDNA and Ku70. MBP pull-down assays revealed a strong interaction between PA^N144A^ and ΔC (Fig. 5G). Co-IP experiments also revealed that like EGFP-PA alone, co-expression of EGFP-PA^N144A^ and EGFP-ΔC augmented the association between Ku70 and DNA-PKcs (Fig. 5H). Importantly, NHEJ reporter assays demonstrated that while overexpressing PA^N144A^ or ΔC alone had no effect on NHEJ efficiency, co-expressing PA^N144A^ and ΔC could significantly enhance the NHEJ efficiency to a level comparable to full-length PA/PB (Fig. 5I and Supplementary Fig. S8E). In sum, by oligomerization mediated by the N-terminus of the HD, PRRX1 could simultaneously associate with Ku and dsDNA to facilitate NHEJ (Fig. 5J).

### PRRX1 contributes to the maintenance of genome stability

**Figure 6.**
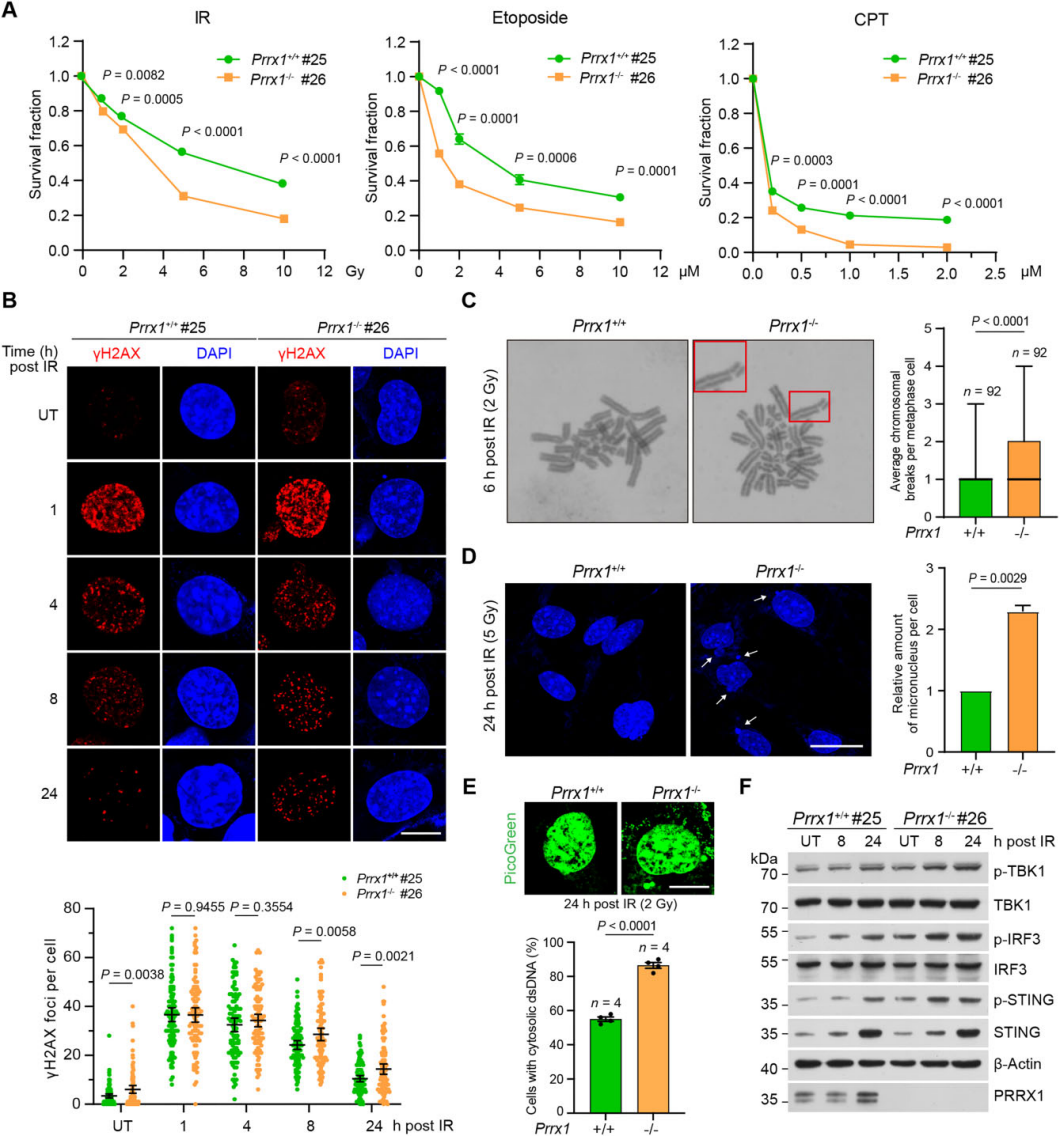
PRRX1 maintains genomic integrity. (**A**) Immortalized WT and *Prrx1*^−/−^ MEF cells were treated with indicated doses of IR, ETP (72 h), or CPT (72 h), and cell survival rates were analyzed by CCK-8 assays (*n*= 3 independent experiments). Data show the mean ± SEM and differences between two different groups were compared using the unpaired, two-tailed Student’s *t*-test. (**B**) WT and *Prrx1*^−/−^ MEF cells were treated with 5 Gy irradiation followed by immunofluorescence (top panel) and quantification of γH2AX foci (bottom panel) at indicated timepoints. Data are mean ± 95% confidence interval (CI). Statistical analysis was performed using two-tailed unpaired *t-*tests. Each point represents one cell; 100 cells quantified in each group were obtained from two independent experiments. UT, untreated. Scale bars, 10 μm. (**C**) Irradiated WT and *Prrx1*^−/−^ MEF cells were measured with chromosome/chromatid breaks (magnification 1000×). The boxes show representative breaks. The right chart indicates the quantification. *n*= 92 cells per genotype, pooled from three independent experiments. Solid lines show median values, and the whiskers extend from the minimum to maximum value. Statistical analysis was performed using two-tailed unpaired *t*-tests. (**D**) Representative DAPI images (left panel) and quantifications (right panel) of micronucleus in irradiated MEF cells. The arrows indicate the micronucleus. Data are mean ± SEM. Statistical analysis was performed using two-tailed unpaired *t*-tests. Scale bar, 25 μm. (**E**) Representative PicoGreen images (top panel) and quantifications (bottom panel) of cytoplasmic dsDNA in irradiated MEF cells. Data are mean ± SEM. Statistical analysis was performed using two-tailed unpaired *t*-tests. Each point represents one experiment; 200 cells quantified in each group were obtained from four independent experiments. Scale bar, 10 μm. (**F**) WT and *Prrx1*^−/−^ MEF cells were treated with 2 Gy irradiation and harvested at indicated times. Cell lysates were immunoblotted with indicated antibodies.

Having showed that PRRX1 facilitates recruitment of Ku70/80 to DSBs and promotes NHEJ, we proceeded to investigate whether PRRX1 plays a role in the DSB repair. Immortalized KO MEFs exhibited increased sensitivities to X-ray IR and several DNA-damaging agents, including the topoisomerase II inhibitor ETP and the topoisomerase I inhibitor CPT (Fig. 6A and Supplementary Fig. S9A). WT and KO MEFs were treated with IR followed by γH2AX immunofluorescence to determine whether PRRX1 affects DNA repair efficiency. *Prrx1*^−/−^ MEFs displayed significant higher numbers of γH2AX foci at late timepoints (8 h and 24 h post IR), implying that the loss of PRRX1 impairs DSB repair (Fig. 6B and Supplementary Fig. S9B). Previous studies have shown that deficiency in DSB repair leads to persistent DNA damage, micronucleus formation, and leakage of damaged nuclear DNA into the cytoplasm, which activates the innate immune response [55–57]. Notably, we observed that *Prrx1*^−/−^ MEFs had an increased frequency of chromosomal breaks, micronucleus, and cytosolic dsDNA compared with those from WT littermates (Fig. 6C–E). Consequently, irradiated *Prrx1*^−/−^ MEFs displayed increased levels of phosphorylated TBK1, IRF3, and STING, indicating enhanced response to damaged DNA (Fig. 6F and Supplementary Fig. S9C). Together, these results underscore the crucial role of PRRX1 in promoting DSB repair *via* NHEJ, highlighting its significance in enhancing cellular resistance to genotoxic stress and maintaining genomic stability.

### Low expression and pathogenic mutation of PRRX1 are associated with genomic instability and defective NHEJ repair

**Figure 7.**
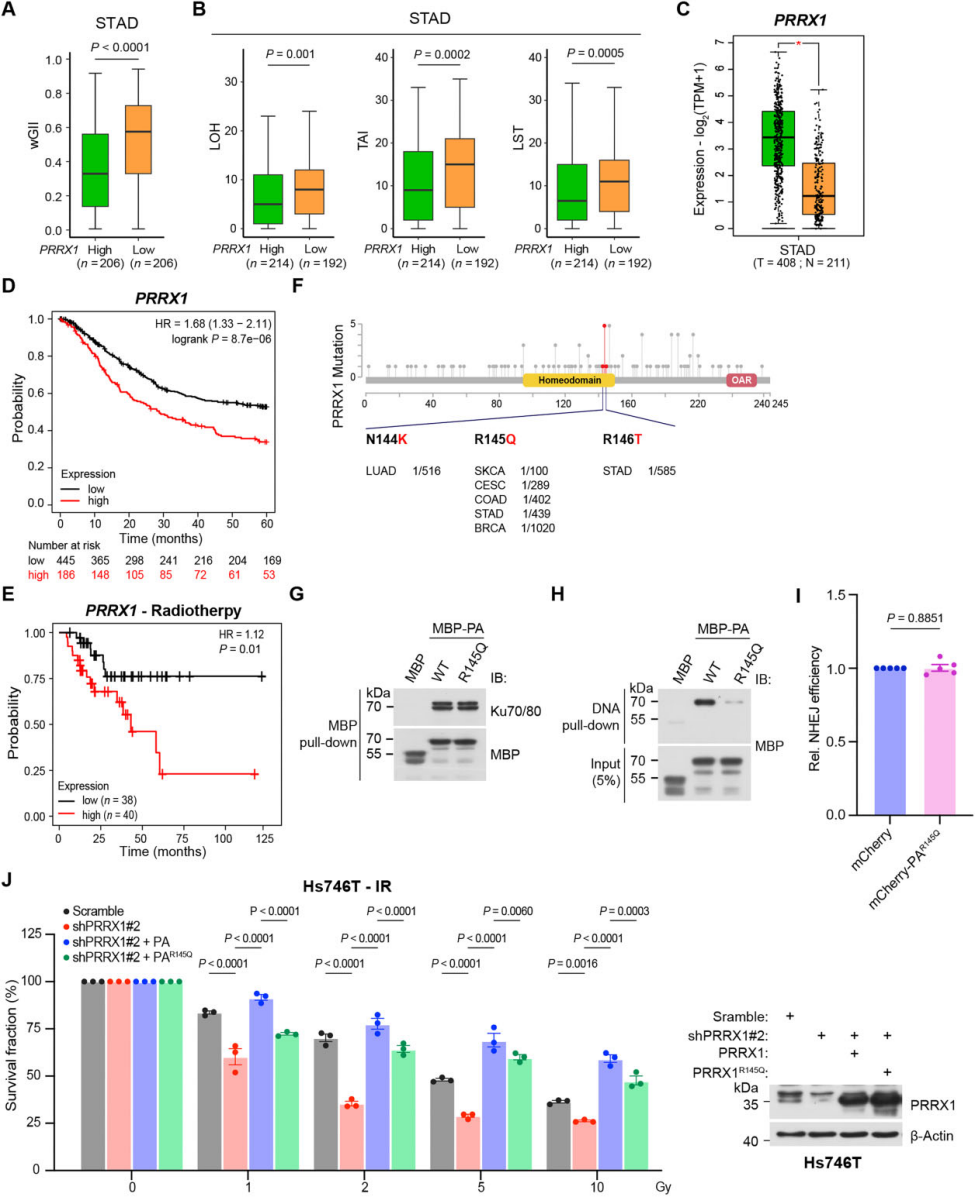
PRRX1 loss or mutation causes genome instability and NHEJ defect. The genomic instability signatures (the wGII) (**A**) and the genomic scar signatures (LOH, LST, and TAI) (**B**) were analyzed using the TCGA STAD cohort. The data were grouped based on *PRRX1* expression levels using the median cutoff. The outline of each box represents the 25th and 75th percentiles, with solid lines indicating median values. The whiskers extend to the most extreme data points within 1.5 × the interquartile range. Statistical significance was determined using a two-tailed Mann–Whitney Wilcoxon test. (**C**) Expression difference of *PRRX1* in STAD samples. The data were based on the TCGA database and retrieved *via* the GEPIA2 website. Kaplan–Meier survival curves for overall survival of STAD patients with high [top 30 (**D**) or 50 (**E**) percentile] or low [bottom 70 (panel D) or 50 (panel E) percentile] *PRRX1* expression. Data were based on the TCGA database. The HR (i.e. hazard ratio) and log-rank *P*-value were indicated in each panel. (**F**) The diagram illustrating PRRX1 mutations identified in various tumors. Data were retrieved from the ICGC database. Numbers represent case frequencies. (**G**) MBP pull-down assays using recombinant MBP-PA and MBP-PA^R145Q^ to co-purify His-Ku70/80. Co-purified products were immunoblotted with indicated antibodies. (**H**) DNA pull-down of dsDNA with recombinant MBP-PA and MBP-PA^R145Q^. Co-purified products were immunoblotted with an anti-MBP antibody. (**I**) EJ5-GFP NHEJ reporter assays of U2OS cells overexpressing mCherry or mCherry-PA^R145Q^ (*n*= 5 biologically replicates). Statistical analysis was performed using two-tailed unpaired *t*-test. (**J**) Left panel: Survival analyses of Hs746T STAD cells with indicated treatment. Statistical analysis was performed using two-way ANOVA. Right panel: Cell lysates were immunoblotted with indicated antibodies.

In cancer cells, dysregulated DDR mechanisms often lead to resistance or sensitivity to chemotherapy and radiotherapy [12]. Having illustrated the roles and mechanisms of PRRX1 in NHEJ, we then examined how PRRX1 expression and mutations occurring in tumors could affect NHEJ. We analyzed the wGII [39], a widely used signature of genomic instability, in STAD samples from the TCGA database. The data revealed that tumors with low PRRX1 expression displayed higher wGII (Fig. 7A). Concordantly, STAD samples with lower PRRX1 expression displayed significant higher scores for three independent genomic scar signatures [41]—LOH, LST, and TAI (Fig. 7B), suggesting an increased burden of chromosomal instability with low PRRX1 expression. Furthermore, STAD tumor samples exhibited significantly higher expression levels of *PRRX1* compared with nontumor tissues (Fig. 7C). High *PRRX1* expression was associated with poorer overall survival (Fig. 7D) and correlated with a worse prognosis in STAD patients undergoing radiotherapy (Fig. 7E).

By analyzing the International Cancer Genome Consortium (ICGC) database, a series of mutation sites on *PRRX1* were identified in patients with various cancers (Fig. 7F). Among them, properties of PRRX1^R145Q^, found in cancer samples including STAD, breast cancer and colon cancer, were inspected. Strikingly, PRRX1^R145Q^ could bind to Ku70/80 but not dsDNA (Fig. 7G and H). Consistently, PRRX1^R145Q^ was unable to enhance NHEJ repair (Fig. 7I), implying that tumors harboring PRRX1^R145Q^ might be more sensitivity to DNA damaging agents. We then studied how PRRX1 affects the response to IR in PRRX1 abundant Hs746 STAD cells, a preclinical model representing the “mesenchymal-like” or “genome stability” subtype of STAD [58–60]. Loss of PRRX1 caused hypersensitivity to irradiation, which was fully rescued by overexpressing WT PRRX1 but only partially rescued by the PA^R145Q^ mutant (Fig. 7J). Together, given its role in facilitating NHEJ, PRRX1 may serve as a potential target to enhance radiotherapy effectiveness for specific subtypes of STAD.

### The peptide disrupting PRRX1 oligomerization blocks NHEJ

**Figure 8.**
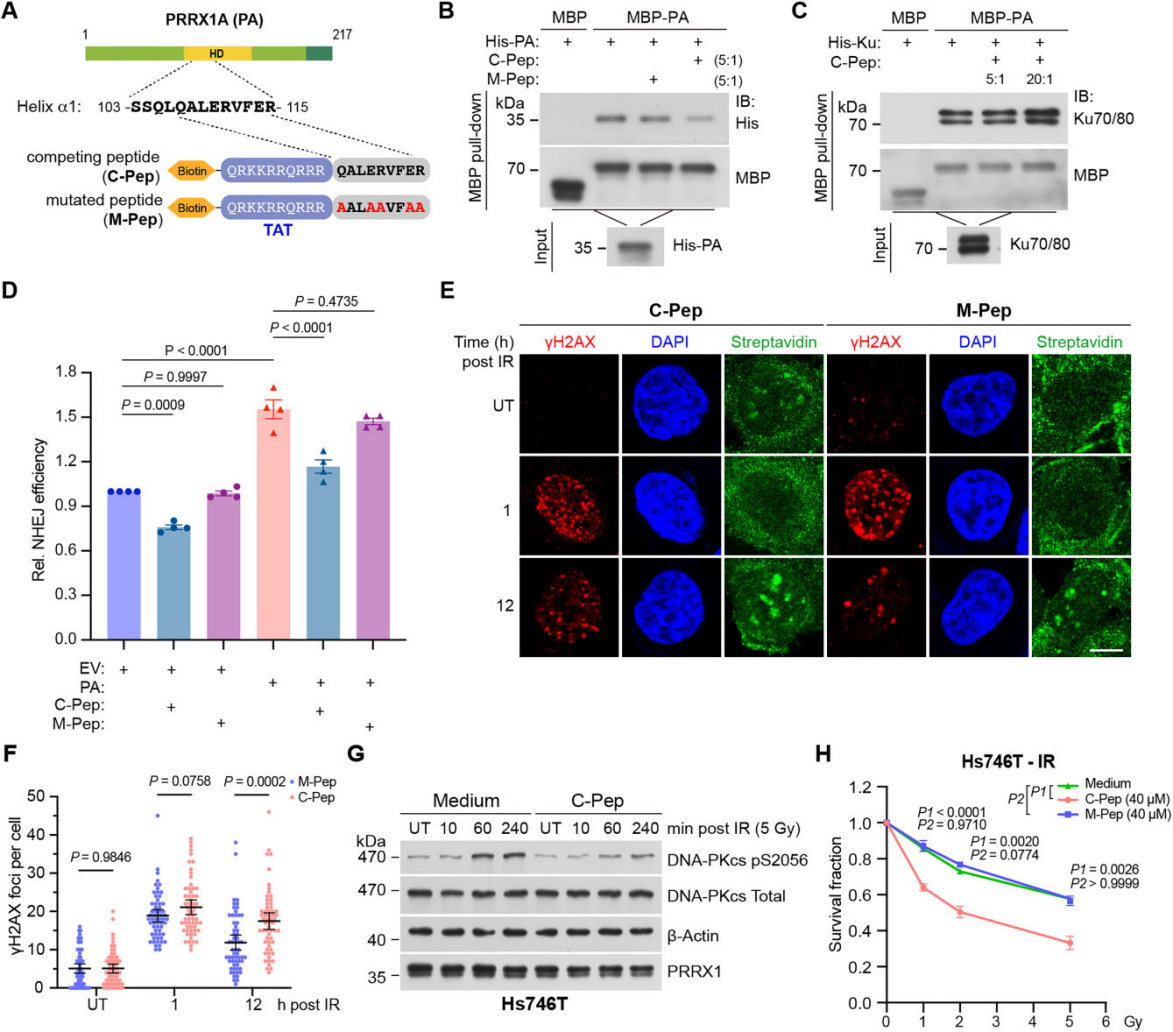
The peptide disrupting PRRX1 oligomerization blocks NHEJ. (**A**) A diagram depicting the peptide design (competing and mutated peptides) for perturbing PRRX1 oligomerization. The N-terminal TAT peptide was used to facilitate cell entry. MBP pull-down assays using recombinant MBP-PA to co-purify His-PA (**B**) and His-Ku70/80 (**C**) with the presence of indicated peptides. Co-purified products were immunoblotted with indicated antibodies. The indicated proportions (peptides: PRRX1) represent the ratio of molar concentration. (**D**) EJ5-GFP NHEJ reporter assays of U2OS cells overexpressing EV or PA with the presence of indicated peptides. *n*= 5 biologically replicates. Statistical analysis was performed using two-way ANOVA. (**E**) Hs746T cells were irradiated and treated with 40 μM indicated peptides. Represented immunofluorescence images of γH2AX, DAPI and biotinylated peptides were shown. UT, untreated. Scale bars, 10 μm. (**F**) Quantification of γH2AX foci in panel (E). Data are mean ± 95% CI. Each point represents a cell; 60 cells quantified in each group were obtained from two independent experiments. Statistical analysis was performed using two-tailed unpaired *t-*tests. (**G**) Hs746T cells were treated with 40 μM peptides and harvested at indicated time post IR. Cell lysates were immunoblotted with indicated antibodies. (**H**) Survival analysis of Hs746T cell with indicated treatment. Statistical analysis was performed using two-way ANOVA.

We finally sought to modulate NHEJ efficiency by disrupting the oligomerization of PRRX1. The competing nine-amino acid peptide, targeting the N-terminal helix α1 of PRRX1’s HD, was synthesized and placed at the carboxyl end of the biotinylated TAT peptide to promote nuclear entry. A five-amino acid mutated peptide was also synthesized to serve as the control (Fig. 8A). The MBP pull-down assay revealed that the competing peptide, but not the mutated peptide greatly compromised the self-binding of MBP- and His-PRRX1 (Fig. 8B), while both peptides exhibiting no effect on the interaction between MBP-PRRX1 and Ku70/80 (Fig. 8C). Importantly, the competing peptide reduced NHEJ efficiency in both mock and PRRX1-overexpressing U2OS cells (Fig. 8D), and the number of γH2AX foci was significantly higher in cells treated with competing peptide compared with those treated with the mutant peptide (Fig. 8E and F). Additionally, the presence of the competing peptide attenuated the phosphorylation of DNA-PKcs at serine 2056 in Hs746T cells following IR treatment (Fig. 8G). Consistently, the competing rather than the mutant peptide, significantly undermined the survival of irradiated Hs746T cells (Fig. 8H). Thus, disrupting the oligomerization of PRRX1 may represent a potential therapeutic strategy for treating STAD.

## Discussion

The initiation of NHEJ requires the attachment of Ku70/80 to the DSB ends, enabling the formation and activation of the DNA–PK holoenzyme. However, many questions remain regarding how Ku70/80 is attached and stabilized at broken DNA ends. In this study, we unveiled a novel role for PRRX1, conventionally recognized as a HD transcription factor, in promoting the accumulation of Ku70/80-DNA-PKcs on DSBs. PRRX1 oligomerizes to enable its simultaneous interactions with both dsDNA and the SAP domain of Ku70, thereby enhancing Ku anchoring at DSBs to stabilize DNA–PK for efficient NHEJ repair (graphical abstract). The study advances our understandings of the molecular mechanisms underlying NHEJ and offers novel insights into cancer therapeutics.

### Oligomerized PRRX1 modulates the dynamics of the DNA–PK complex at DSB sites

The ring-shaped Ku70/80 binds dsDNA by contacting a few DNA backbone phosphates, allowing ATP-independent sliding along DNA. Structural studies show the SAP domain in Ku70/80, a flexible DNA aperture component, facilitates initial Ku-DNA contact [61], acting as a “linker” connecting NHEJ machinery with co-factors. The Ku80 C-terminal stabilizes the DNA–PK complex but is not essential for its formation. The Ku70 SAP domain is critical for Ku70/80 association with dsDNA *in vivo* [19, 20], but its role in localizing Ku70/80 to damaged DNA ends remains unresolved. Recent findings suggest that the Ku70 SAP domain limits Ku’s lateral movement along DNA and is essential for repairing exogenous DSBs [62, 63].

Several factors regulate NHEJ by interacting with Ku heterodimers. For instance, ZNF384 acts as an adapter binding to DNA and Ku70 in a PARP1-dependent manner, promoting Ku70/80 accumulation at DSBs [28]. VAV2 facilitates Ku heterodimer formation by binding Ku70 and Ku80, enhancing NHEJ [64]. Methylation of Ku70 by SMYD2 promotes DNA–PK complex assembly [29]. Conversely, FOXL2 and LRRC31 inhibit Ku70/80 heterodimer or DNA–PK complex formation, weakening NHEJ repair [37, 65]. Interestingly, the Ku70 SAP domain associates with several HD proteins. Schild-Poulter *et al.* identified Ku70 interactions with HD proteins, including HOXC4, HOXD4, and OCT2, in yeast [66], though their role in DNA damage was unclear. Later, Rubi *et al.* found elevated HOXB7 expression in human breast epithelial cells enhanced resistance to DNA damage, suggesting HD proteins might assist DNA repair as Ku70 partners [67]. Mechanistic studies confirmed that HOXB7 binds to Ku70, Ku80, and DNA-PKcs *via* its HD domain, linking DNA damage resistance to these interactions. CDX2, another HD protein, binds Ku70 through its HD domain in colorectal cancer cells but inhibits DNA–PK activity [68]. While Ku70 binding appears to be a common feature of HD proteins, its precise role in NHEJ repair and the underlying mechanisms remain insufficiently explored.

In this study, we discovered that HD protein PRRX1 binds to the SAP domain of Ku70. Importantly, depletion of PRRX1 markedly reduced the enrichment of Ku70/80 on the chromatin and compromised NHEJ, whereas the opposite effect was observed upon PRRX1 overexpression. In particular, PRRX1, despite being relatively small, simultaneously interacts with the SAP domain of Ku70 and dsDNA through its NRR motif within the HD. PRRX1 achieves this by forming oligomers *via* its N-terminal helix α1 (107–115 aa) in the HD domain. Of note, the motif essential for PRRX1 oligomerization is unique to PRRX1 and not found in other HD proteins, making it a potential target for improving cancer treatment. Indeed, the competing peptide that disrupts PRRX1 oligomerization blocked NHEJ and hampered the survival of irradiated STAD cells. In addition, we also revealed that PRRX1-specific sequences at both N-terminal and C-termnial parts are involved in its association with Ku70 and dsDNA, as well as oligomerization.

### PRRX1 senses and accumulates at DNA damage sites

LMI studies revealed that both endogenous and exogenous PRRX1 rapidly accumulate at DNA damage sites. Intriguingly, this accumulation occurs irrespective of known DNA damage sensors, including PARP1, Ku, and the MRN complex, as well as upstream kinases such as ATM and DNA-PKcs. Instead, PRRX1 directly binds to various forms of dsDNA with random sequences but not ssDNA *in vitro*. Moreover, PRRX1 was found to be associated with chromatin along with Ku70/80 in untreated, irradiated, and ETP treated cells. Further investigations are needed to determine whether PRRX1 exhibits a preference for specific regions or sequences in terms of chromatin and DSB association.

The two isoforms of PRRX1 have different C-terminal ends, which are seemingly not required for its oligomerization or DNA or Ku70 binding. Moreover, both exogenous PA and PB exhibited the same kinetics in the recruitment to DSBs. However, we have revealed a few discrepancies of the two isoforms. First, DNA pull-down assays demonstrated that the shorter PA had a stronger affinity for dsDNA. We speculate that potential co-factors or post-translational modifications of PRRX1 in the cellular context might compensate for the difference in their dsDNA affinity. Second, the sucrose gradient separation assay indicated that the two isoforms were enriched in adjacent and overlapping compartments. Third, the oligomerization efficiency of PA is higher than PB. Fourth, the NHEJ efficiency in cells overexpressing PA was around 10% higher than those overexpressing PB. Further investigation is needed to determine whether the two isoforms are differentially utilized in specific cellular or stress contexts. Additionally, the possibility that PA and PB function as heterodimers should be explored.

### PRRX1 is associated with defective NHEJ repair in certain cancer types

PRRX1, traditionally recognized as a transcription factor, plays essential roles in specification of mesodermal and neural crest lineages, as well as in the tumorigenesis and metastasis of cancer cells. We have previously demonstrated that the NRR motif is crucial for PRRX1’s transcriptional function [46]. Interestingly, PRRX1 could transcriptionally maintain the expressions of DDR genes including *ATM*, *ATR*, *BRCA1*, *PRKDC*, and *XRCC1*, in PANC1 pancreatic cancer cells [34]. Here, we unveiled the transcription-independent role of PRRX1 in promoting NHEJ. As anticipated, the conserved NRR motif (144–146 aa) within the HD domain is required for PRRX1’s DNA association, and other domains could facilitate the binding, likely by maintaining the proper conformation.

We have meticulously examined the impact of NRR mutations, including the R145Q mutant, identified in various cancers such as STAD, on Ku- and dsDNA-binding, as well as oligomerization. These mutated PRRX1 losses the ability to associate with either Ku70 or dsDNA, ultimately leading to the defect of facilitating NHEJ. In STAD, low expression levels of *PRRX1* associate with genomic instability, while high expression levels of PRRX1 correlate with poor prognosis and IR resistance. It remains to be investigated whether PRRX1 has a transcription-independent role in regulating cancer microenvironment, such as cancer-associated fibroblasts [32], and how PRRX1-mediated regulation of NHEJ efficiency could influence responses to combination therapies, particularly those involving immune checkpoint inhibitors.

## Supplementary Material

gkaf200_Supplemental_File

## Data Availability

The RNA-seq data of MEFs have been deposited in the GEO database (RRID: SCR_005012) with accession number GSE265857 (access link: https://www.ncbi.nlm.nih.gov/geo/query/acc.cgi?acc=GSE265857; password: ehkvascqdnmlbgh). The MS data have been deposited to the ProteomeXchange Consortium (https://proteomecentral.proteomexchange.org; RRID: SCR_004055) *via* the iProX repository [69] with the dataset identifier PXD051766 (access link: https://www.iprox.cn/page/PSV023.html;?url=1714117094993dGyS; password: a509). All data are publicly available from the date of publication. Any additional information required to reanalyze the data in this paper is available from the authors upon rational request.
