# Supplementary material for: Homeodomain protein PRRX1 anchors the Ku heterodimers at DNA double-strand breaks to promote nonhomologous end-joining
Source: Nucleic Acids Res. 2025 Mar 20;53(6):gkaf200. doi: 10.1093/nar/gkaf200 (PMC11925728; doi:10.1093/nar/gkaf200)
Supplement: gkaf200_Supplemental_File [file gkaf200_supplemental_file.pdf]

Supplementary Data

Homeodomain protein PRRX1 anchors the Ku heterodimers at DNA double-strand breaks to promote non-homologous end-joining

Yan Wang[1,2,#], Fuyuan Shen[1,2,#], Chen Zhao[1,2], Jiali Li[1,2], Wen Wang[1,2], Yamu Li[3], Jia Gan[1,2], Haojian Zhang[2], Xuefeng Chen[2], Qiang Chen[2], Fangyu Wang[1,2,*], Ying Liu[1,2,*], Yan Zhou[1,2,*]

[1]Department of Neurosurgery, Medical Research Institute, Zhongnan Hospital of Wuhan University, Wuhan University, Wuhan, China
[2]Frontier Science Center of Immunology and Metabolism, Wuhan University, Wuhan, China
[3]The First Affiliated Hospital of Henan University
[#]Equal contribution
*To whom correspondence should be addressed: fangyu.wang@whu.edu.cn; y.liu@whu.edu.cn; yan.zhou@whu.edu.cn

Supplementary Figure S1 - S9 Page 2 - 10

Supplementary Table S1 - S3 Page 11 - 20

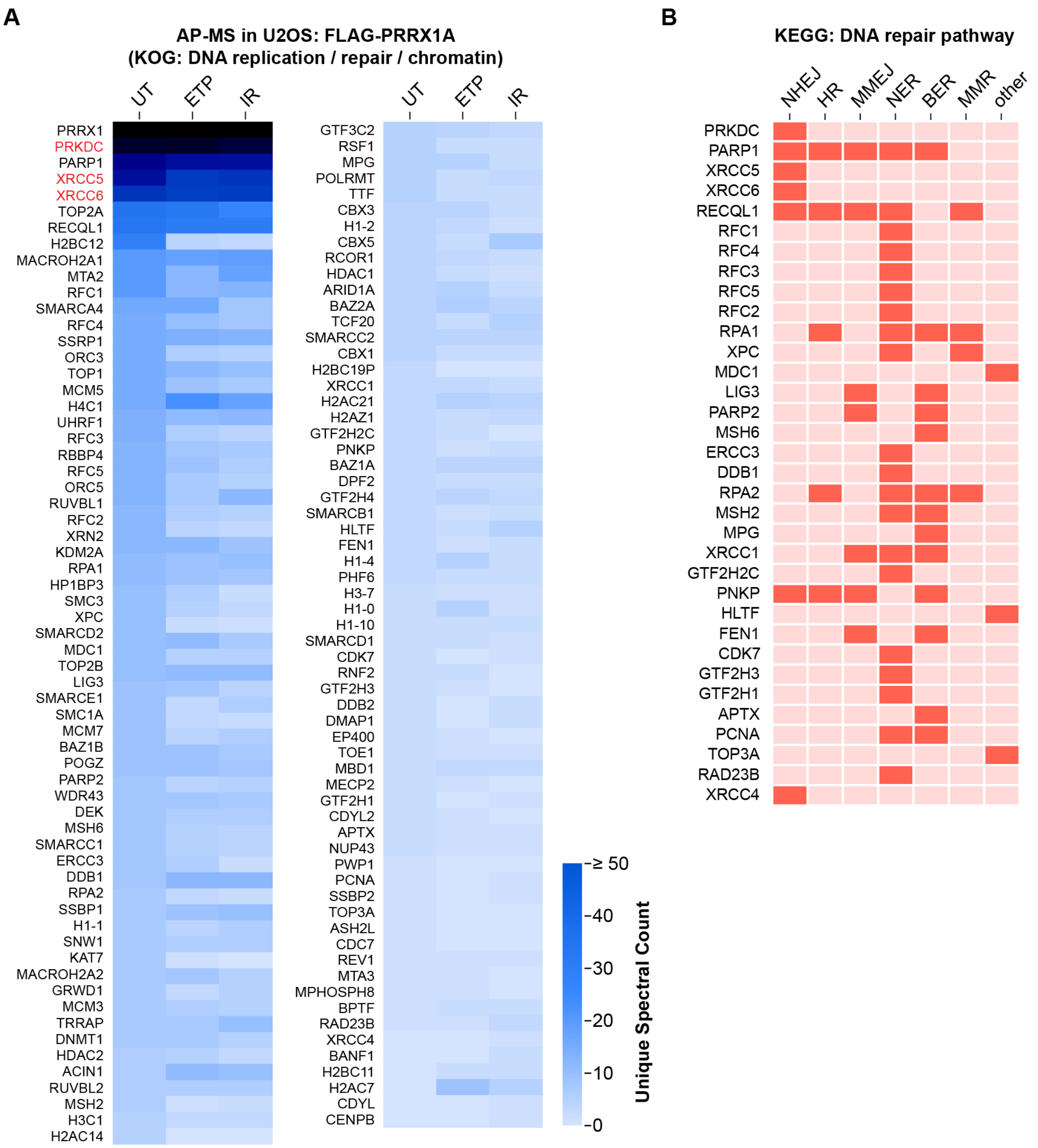

Figure S1. PRRX1 associates with components of the DNA repair machinery and chromatin.

(**A**) Heat map depicting the unique spectral counts of proteins involved in DNA replication, repair, or chromatin that were co-purified with FLAG-PRRX1A. Components of the DNA-PK complex are highlighted in red. (**B**) Components of the DNA damage repair pathways identified in (A) were categorized using the KEGG database. NHEJ, nonhomologous end joining; HR, homologous recombination; MMEJ, microhomology-mediated end joining; NER, nucleotide excision repair; BER, base excision repair; MMR, mismatch repair.

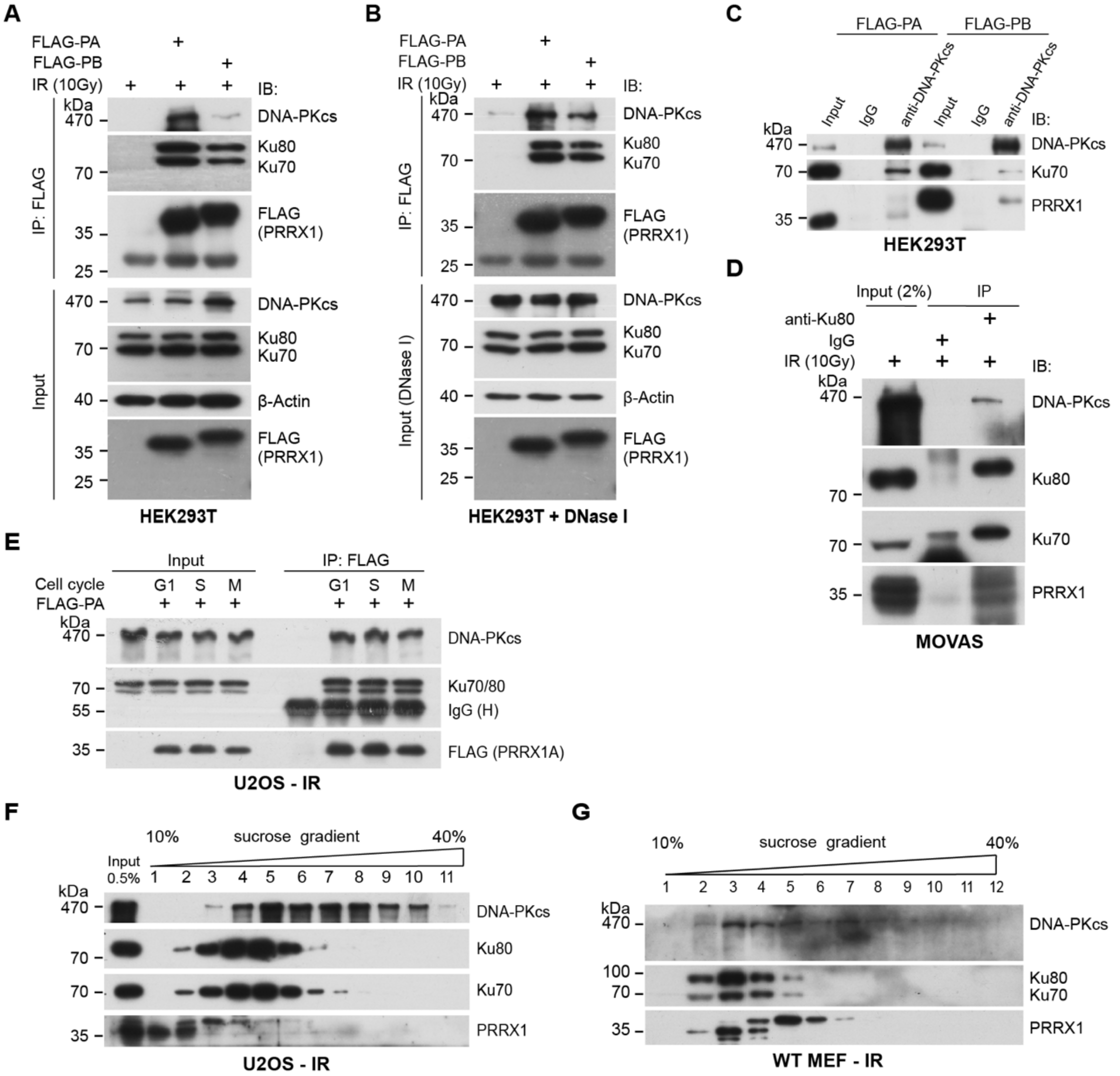

Figure S2. PRRX1 interacts with components of the Ku-DNA-PKcs complex.

(**A**-**C**) FLAG-PA or FLAG-PB overexpressing HEK293T cells were irradiated (A and C) and treated with DNase I (B). Co-immunoprecipitants using anti-FLAG beads (A and B) or the anti-DNA-PKcs antibody (C) were immunoblotted with indicated antibodies. (**D**) Lysates of irradiated MOVAS cell were precipitated with anti-Ku80 antibody followed by immunoblotting with indicated antibodies. (**E**) U2OS cells overexpressing FLAG-PA were synchronized at indicated cell cycle phases using thymidine or colchicine. Co-immunoprecipitants using anti-FLAG beads were immunoblotted with indicated antibodies. (**F**, **G**) Lysates of irradiated U2OS (F) or MEF (G) cells were separated by sucrose gradient centrifugation followed by immunoblotting with indicated antibodies. All experiments were performed for at least three times and representative blots were shown.

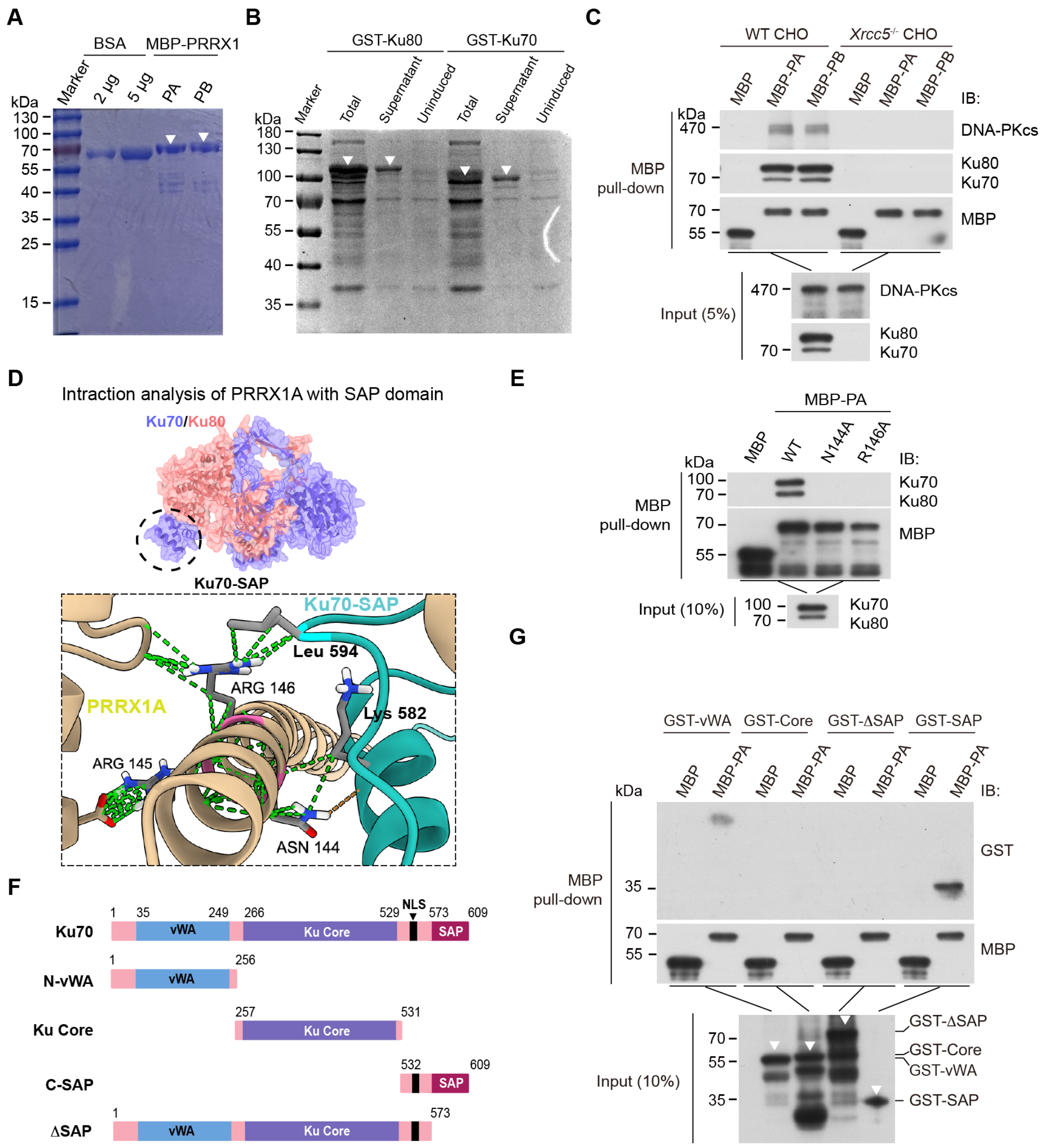

Figure S3. PRRX1 directly binds to the SAP domain of Ku70.

(**A**, **B**) *In vitro* expressed and purified MBP-PRRX1(A), GST-Ku70 and GST-Ku80 (B) were stained with Coomassie Brilliant Blue. (**C**) MBP pull-down assays using recombinant MBP-tagged PRRX1 to co-purify Ku70/80 and DNA-PKcs using lysates of wild-type and *Xrrc5*$^{-/-}$ CHO cells. Co-purified lysates were immunoblotted with indicated antibodies. (**D**) H-bonds and contacts analysis of interaction between PRRX1 with the SAP domain of Ku70. (**E**) MBP pull-down assays using wild-type and mutated PRRX1A to co-purify His-tagged Ku70/80 heterodimer. Co-purified products were immunoblotted with indicated antibodies. (**F**) Diagram depicting wild-type and truncations of human Ku70. Numbers indicate amino acid residues. (**G**) MBP pull-down assays using MBP-PRRX1 to co-purify GST-Ku70 truncations. Co-purified products were immunoblotted with indicated antibodies. White triangles indicate bands with predicted sizes. IB, immunoblotting. All experiments were performed for at least three times and representative blots were shown.

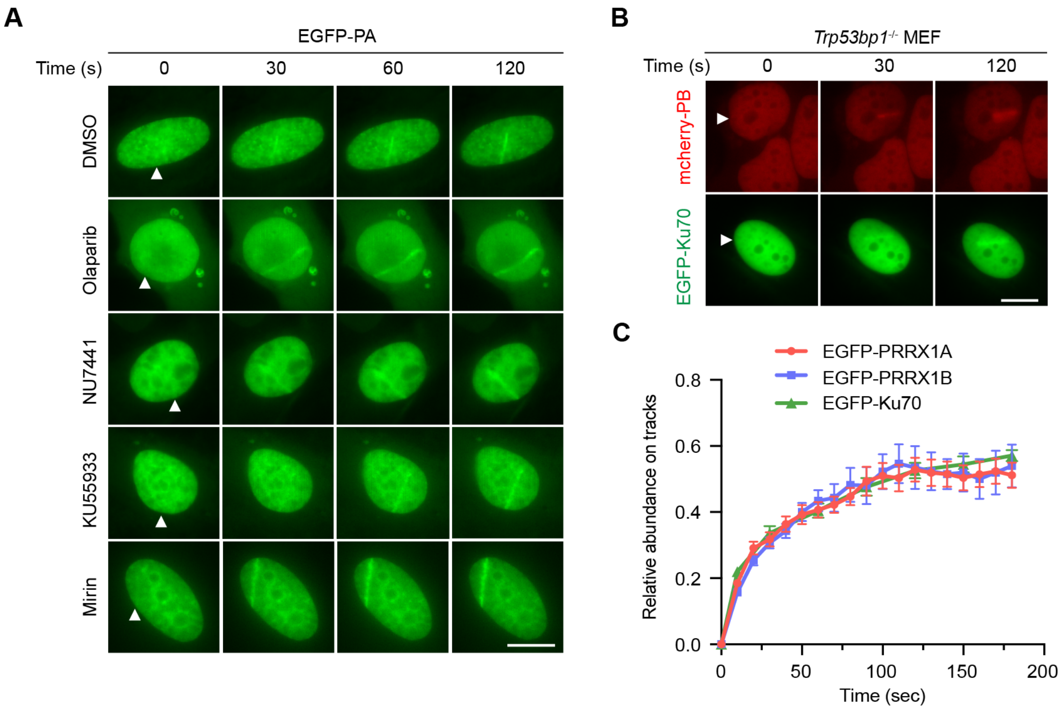

Figure S4. PRRX1 accumulation at DNA damage sites is independent of PARP, DNA-PKcs, ATM, MRE11, or 53BP1.

(**A**) U2OS cells were transfected with EGFP-PRRX1A (PA) and treated with DMSO, 10 µM PARP inhibitor (Olaparib)，2 µM DNA-PK inhibitor (NU7441), 5 µM ATM inhibitor (KU55933), or 50 µM MRE11 inhibitor (Mirin) for 6 h prior to LMI. (**B**) *Trp53bp1*$^{-/-}$ MEF cells were transfected with vectors expressing EGFP-Ku70 and mCherry-PRRX1B and subjected to LMI. Fluorescence images were collected at indicated time points. White triangles indicate irradiated regions. Scale bars, 10 µm. (**C**) The fluorescence intensity of EGFP-PRRX1A/B was compared with that of EGFP-Ku70 on LMI tracks, as shown in Figures 2B and 3E. Data are presented as the mean fluorescence intensity change of irradiated stripes over background per cell (mean ± SEM). PRRX1A (PA), *n* = 20 cells; PRRX1B (PB), *n* = 20 cells; Ku70, *n* = 15 cells.

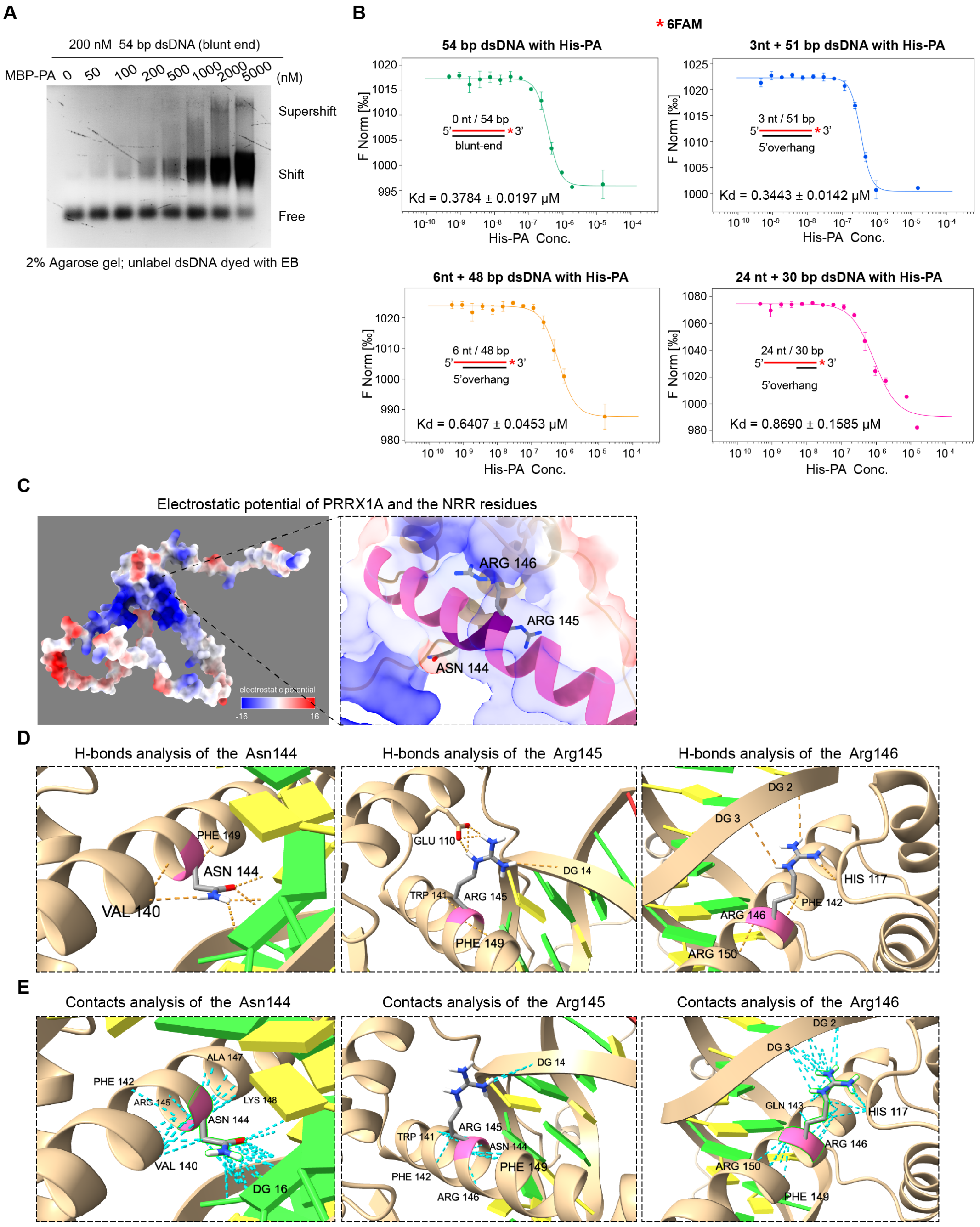

Figure S5. The NRR residues of PRRX1-HD are critical for dsDNA-binding of PRRX1 *in vitro*.

(**A**) EMSA assays of purified MBP-PRRX1A protein with blunt-end dsDNA. Samples were electrophorized in 2% agarose gel and dyed with EB. (**B**) Microscale thermophoresis (MST) analyses of interactions between purified His-PRRX1A with indicated types of dsDNAs. Kd values of binding were shown. (**C**) Electrostatic potential of PRRX1A and the NRR residues. Protein structure files were retrieved from AlphaFold DB. (**D**, **E**) H-bonds (D) and contacts (E) analyses of interactions between PRRX1 residues 144-146 with dsDNA. For (A) and (B), all experiments were performed for at least three times and representative blots were shown.

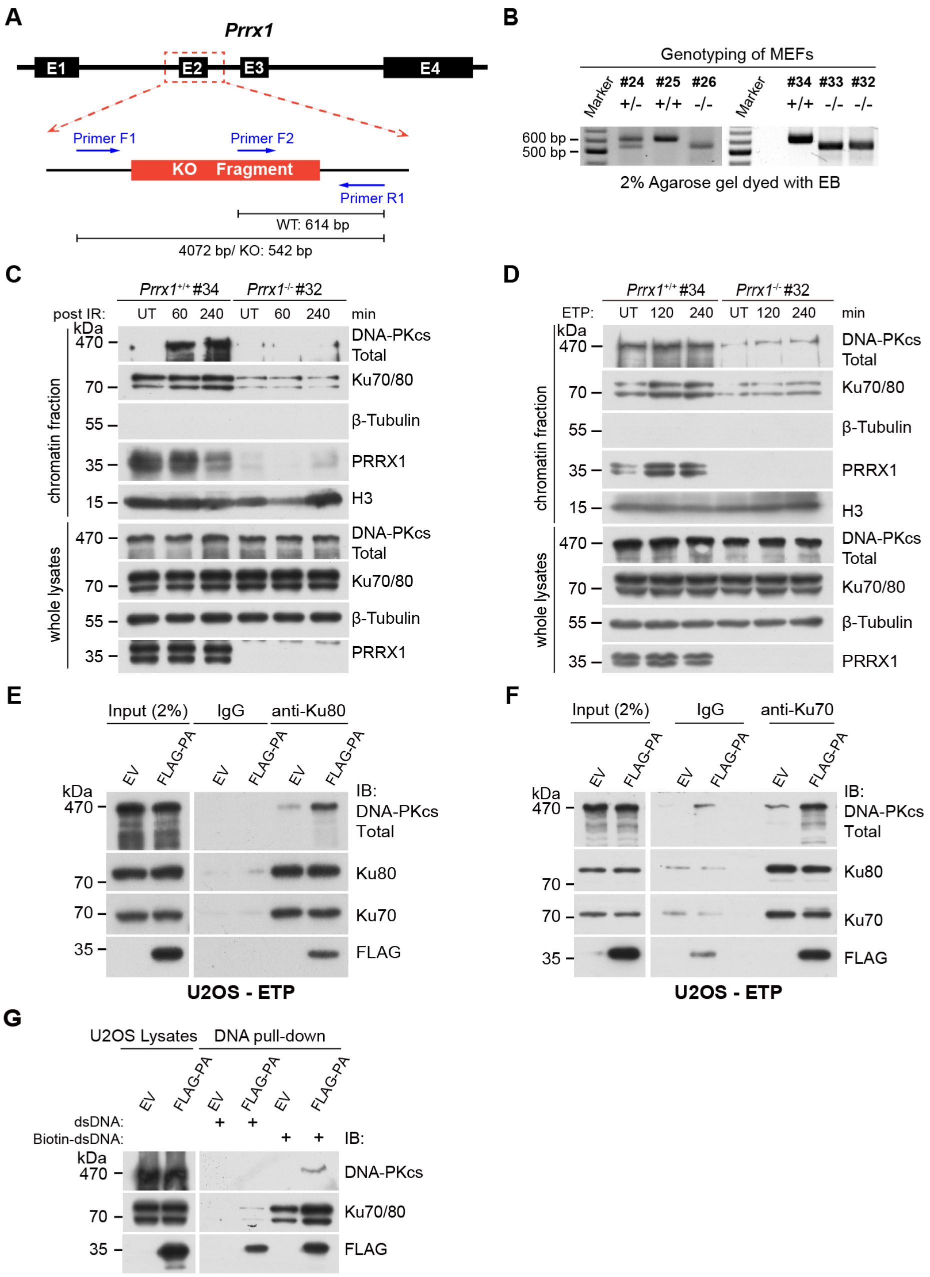

Figure S6. PRRX1 promotes the localization of Ku and the assembly of DNA-PK on chromatin.

(**A**) Diagram depicting the generation of *Prrx1* knockout mice and locations of genotyping primers. (**B**) Electrophoreses of genotyping PCRs for WT and *Prrx1*$^{-/-}$ MEF cells. (**C**, **D**) WT and *Prrx1*$^{-/-}$ MEF cells were treated with 5 Gy irradiation (C) or 10 µM Etoposide (D) at indicated time. Chromatin fractions (top) and whole cell lysates (bottom) were immunoblotted with indicated antibodies. (**E**, **F**) U2OS cells were transfected with EV and FLAG-PRRX1A (PA). Cell lysates were immunoprecipitated with anti-Ku80 (E) and anti-Ku70 (F) antibodies followed by immunoblotting with indicated antibodies. (**G**) U2OS cells were transfected with EV or FLAG-PRRX1A for 36 h. Cell lysates were subjected to DNA pull-downs with blunt-end biotinylated dsDNA followed by immunoblotting with indicated antibodies.

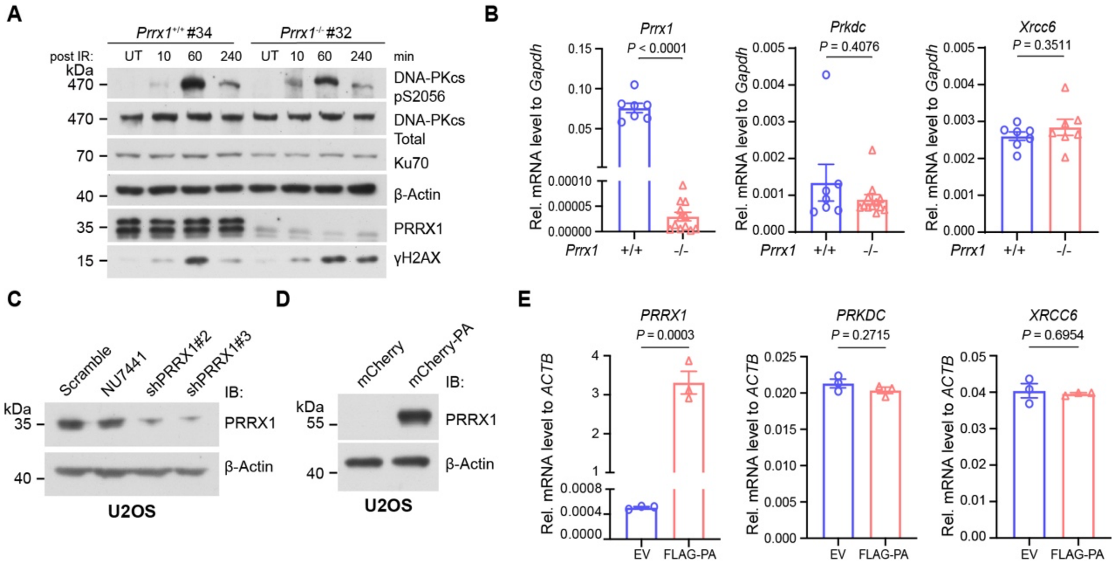

Figure S7. PRRX1 sustains the activation of DNA-PK independently of transcription.

(**A**) WT and *Prrx1*$^{-/-}$ MEF cells were treated with 5 Gy irradiation at indicated times. Cell lysates were immunoblotted with indicated antibodies. (**B**) RT-qPCR analysis depicting relative expressions of *Prrx1*, *Prkdc* and *Xrcc6* of primary MEF cells derived from WT and *Prrx1*$^{-/-}$ littermates. (**C**, **D**) Immunoblotting of U2OS cell lysates used in NHEJ or HR reporter assays. (**E**) RT-qPCR analysis depicting relative expressions of *PRRX1*, *PRKDC* and *XRCC6* of U2OS cells transiently overexpressing FLAG-PRRX1A.

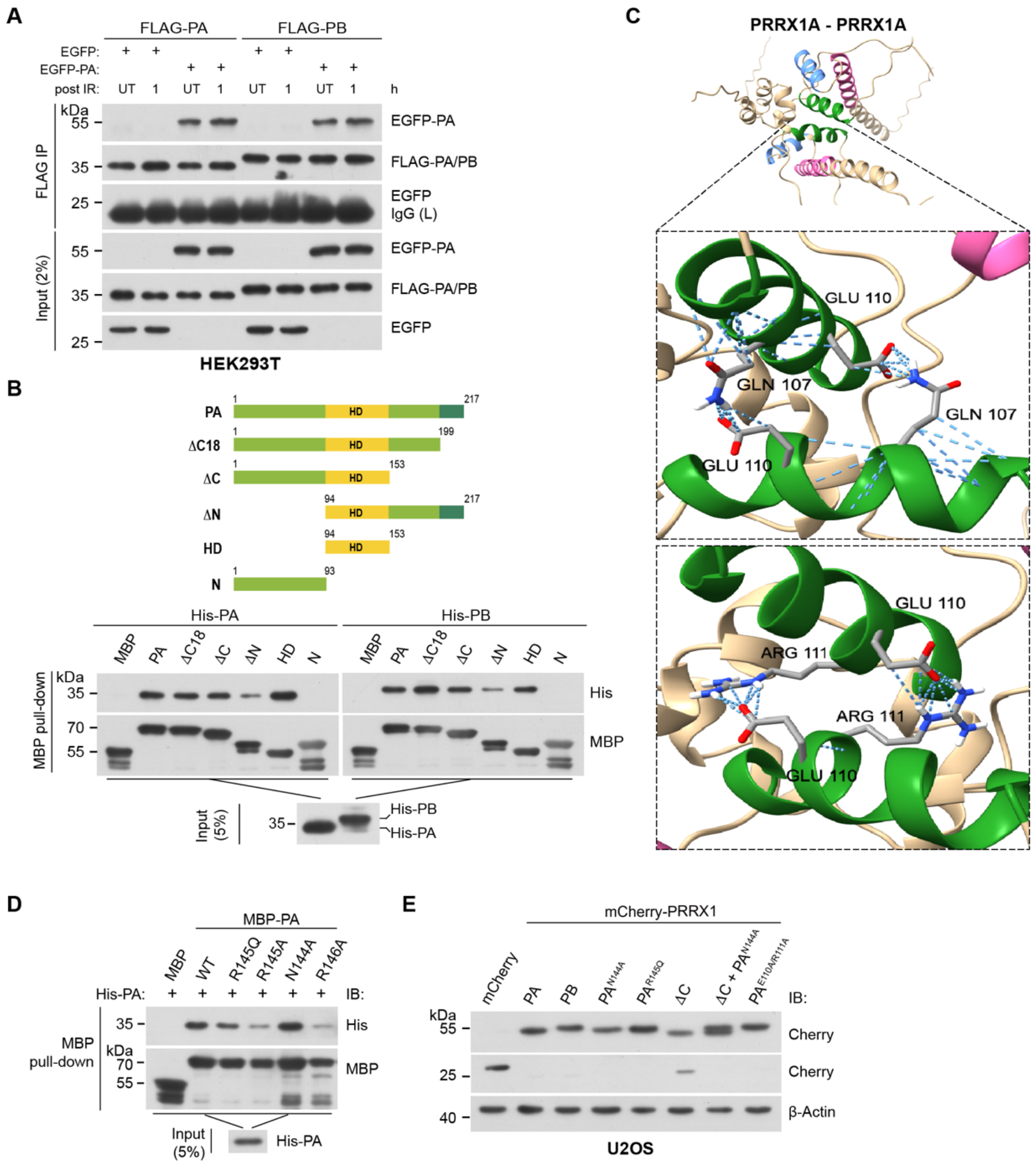

Figure S8. PRRX1 oligomerizes depending on its N-terminus and the homeodomain.
(**A**) HEK293T cells were co-expressed with indicated vectors and subjected to IR. Cell lysates were immunoprecipitated with anti-FLAG beads followed by immunoblotting with indicated antibodies. (**B**) Top: the diagram showing MBP tagged PRRX1A (PA) truncations. Middle and bottom: MBP pull-down assays using recombinant MBP-PA truncations to co-purify His-PA. Co-purified products were immunoblotted with indicated antibodies. (**C**) The docking analysis showing key residues at helix α1 of homeodomain mediates PRRX1 dimerization. (**D**) MBP pull-down assays using recombinant MBP, MBP-PA (WT), and indicated PA mutations to co-purify His-PA. Co-purified products were immunoblotted with indicated antibodies. (**E**) In NHEJ reporter assays, U2OS cells were overexpressed with mCherry tagged PRRX1 mutants. Cell lysates were immunoblotted with indicated antibodies. UT, untreated. IB, immunoblotting.

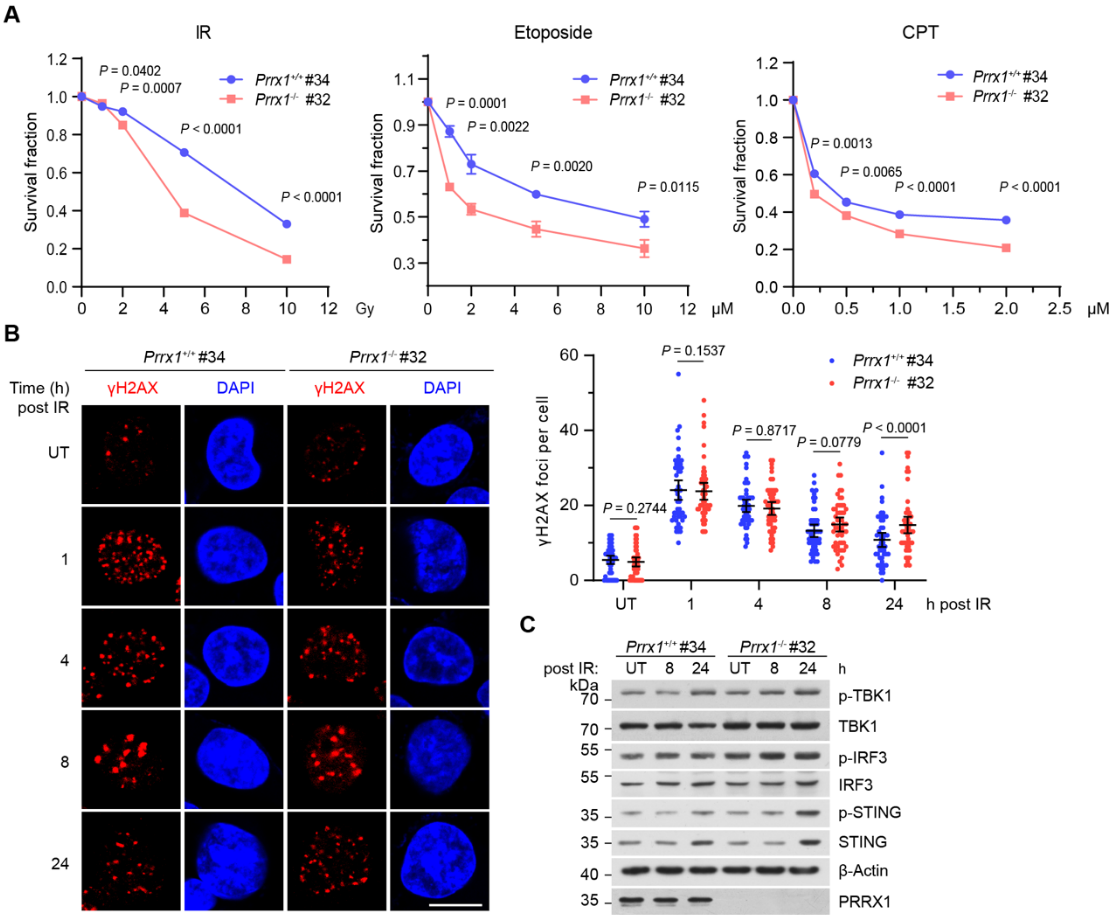

Figure S9. PRRX1 maintains genomic stability in MEF cells.
(**A**) Survival analyses of immortalized WT and *Prrx1*$^{-/-}$ MEF cells after indicated treatment ($n = 3$ biologically independent samples). Data show the mean ± SEM and differences between two different groups were compared using the unpaired, two-tailed Student's *t*-tests. (**B**) WT and *Prrx1*$^{-/-}$ MEF cells were treated with 5 Gy irradiation followed by immunofluorescence (left) and quantification (right) of γH2AX foci at indicated time points. Data are mean ± 95% CI. Statistical analysis was performed using two-tailed unpaired *t*-tests. Each point represents one cell; 100 cells quantified in each group were obtained from two independent experiments. UT, untreated. Scale bars, 10 μm. (**C**) WT and *Prrx1*$^{-/-}$ MEF cells were treated with 2 Gy irradiation and harvested at indicated times. Cell lysates were immunoblotted with indicated antibodies. UT, untreated.

Table S1. The gene list of proteins identified in FLAG-PRRX1 AP-MS

**FLAG-PRRX1A (in GICs)**							
**Accession**	**Gene symbol**	**Coverage %**	**Σ# Proteins**	**Σ# Unique Peptides**	**Σ# PSMs**	**MW [kDa]**	**FA/Control (> 5)**
P78527	*PRKDC*	16.55	1	59	102	468.8	#DIV/0!
P51114	*FXR1*	4.67	3	3	5	69.7	#DIV/0!
Q9UN86	*G3BP2*	7.26	1	3	6	54.1	#DIV/0!
Q12905	*ILF2*	9.74	1	3	9	43.0	#DIV/0!
P17844	*DDX5*	15.96	1	6	18	69.1	#DIV/0!
Q15366	*PCBP2*	24.38	3	3	10	38.6	#DIV/0!
Q15365	*PCBP1*	12.64	1	1	6	37.5	#DIV/0!
P12268	*IMPDH2*	39.49	2	17	39	55.8	#DIV/0!
P00367	*GLUD1*	23.30	2	10	21	61.4	#DIV/0!
Q1KMD3	*HNRNPUL2*	8.30	1	6	10	85.1	#DIV/0!
Q52LJ0	*FAM98B*	18.18	1	3	8	37.2	#DIV/0!
Q9Y224	*C14orf166*	18.85	1	3	7	28.1	#DIV/0!
P04792	*HSPB1*	17.07	1	3	6	22.8	#DIV/0!
P78344	*EIF4G2*	4.63	1	3	6	102.3	#DIV/0!
P42166	*TMPO*	6.77	2	3	6	75.4	#DIV/0!
O75569	*PRKRA*	12.78	1	3	6	34.4	#DIV/0!
Q9Y295	*DRG1*	7.63	1	3	5	40.5	#DIV/0!
Q9NUL3	*STAU2*	8.42	1	3	6	62.6	#DIV/0!
Q99700	*ATXN2*	2.97	1	3	5	140.2	#DIV/0!
Q9HCE1	*MOV10*	2.99	1	2	4	113.6	#DIV/0!
O95782	*AP2A1*	2.05	2	2	4	107.5	#DIV/0!
Q00610	*CLTC*	1.91	1	2	4	191.5	#DIV/0!
P40939	*HADHA*	3.01	1	2	4	82.9	#DIV/0!
P13639	*EEF2*	1.98	1	2	4	95.3	#DIV/0!
Q7L2H7	*EIF3M*	10.70	1	2	4	42.5	#DIV/0!
Q96C36	*PYCR2*	6.25	2	2	4	33.6	#DIV/0!
P51784	*UBP11*	2.28	1	2	4	109.7	#DIV/0!
P47929	*LGALS7*	16.91	1	2	4	15.1	#DIV/0!
P54821	*PRRX1*	59.59	17	21	189	27.3	329.68
P13010	*XRCC5*	51.09	1	31	90	82.7	202.34
P12956	*XRCC6*	39.57	3	25	69	69.8	114.69
Q14444	*CAPRIN1*	15.37	1	9	22	78.3	35.19
P09874	*PARP1*	27.32	1	20	36	113.0	8.23
Q00839	*HNRNPU*	35.76	1	26	106	90.5	8.07
Q8NC51	*SERBP1*	25.25	1	8	20	44.9	7.80
Q9NUQ6	*SPATS2L*	16.85	1	8	17	61.7	6.43
Q13283	*G3BP1*	20.39	1	8	26	52.1	6.04
P11940	*PABPC1*	33.02	5	11	45	70.6	5.77

Q92841	*DDX17*	15.85	2	6	11	72.3	5.61
Q13310	*PABPC4*	25.00	1	6	31	70.7	5.48
O95819	*MAP4K4*	3.95	4	5	11	142.0	5.44
**FLAG-PRRX1B (in GICs)**							
**Accession**	**Gene symbol**	**Coverage %**	**Σ# Proteins**	**Σ# Unique Peptides**	**Σ# PSMs**	**MW [kDa]**	**FB/Control (> 5)**
P78527	*PRKDC*	18.58	1	70	142	468.8	#DIV/0!
P12268	*IMPDH2*	43.00	2	17	48	55.8	#DIV/0!
P17844	*DDX5*	23.13	1	8	29	69.1	#DIV/0!
P51116	*FXR2*	3.86	2	1	6	74.2	#DIV/0!
Q15366	*PCBP2*	18.90	3	3	13	38.6	#DIV/0!
Q9UN86	*G3BP2*	13.69	1	5	11	54.1	#DIV/0!
Q12905	*ILF2*	20.77	1	7	17	43.0	#DIV/0!
Q15365	*PCBP1*	10.11	1	2	6	37.5	#DIV/0!
P13639	*EEF2*	7.46	1	5	9	95.3	#DIV/0!
P51114	*FXR1*	9.34	2	3	10	69.7	#DIV/0!
P00367	*GLUD1*	19.53	2	9	18	61.4	#DIV/0!
P42166	*TMPO*	19.31	2	9	16	75.4	#DIV/0!
Q04837	*SSBP1*	15.54	1	2	4	17.2	#DIV/0!
Q9Y230	*RUVBL2*	13.82	1	5	11	51.1	#DIV/0!
P04792	*HSPB1*	46.34	1	6	12	22.8	#DIV/0!
Q9NUL3	*STAU2*	11.58	1	4	10	62.6	#DIV/0!
Q7L2E3	*DHX30*	2.85	1	3	7	133.9	#DIV/0!
Q96C36	*PYCR2*	11.25	1	4	7	33.6	#DIV/0!
Q9H0D6	*XRN2*	4.63	2	4	10	108.5	#DIV/0!
Q96QR8	*PURB*	14.10	3	4	9	33.2	#DIV/0!
O95793	*STAU1*	3.29	1	1	4	63.1	#DIV/0!
P18085	*ARF4*	9.44	1	1	3	20.5	#DIV/0!
Q9Y224	*C14orf166*	18.85	3	5	9	28.1	#DIV/0!
Q14527	*HLTF*	2.28	1	2	4	113.9	#DIV/0!
Q00610	*CLTC*	4.96	2	6	11	191.5	#DIV/0!
P43003	*SLC1A3*	5.90	1	2	4	59.5	#DIV/0!
Q1KMD3	*HNRNPUL2*	8.43	1	6	11	85.1	#DIV/0!
Q8WWM7	*ATXN2L*	5.86	1	5	8	113.3	#DIV/0!
Q8WU90	*ZC3H15*	10.56	1	4	8	48.6	#DIV/0!
Q99700	*ATXN2*	3.88	1	3	7	140.2	#DIV/0!
Q7Z417	*NUFIP2*	5.04	1	3	6	76.1	#DIV/0!
P41091	*EIF2S3*	9.75	2	4	7	51.1	#DIV/0!
O15397	*IPO8*	4.92	1	4	8	119.9	#DIV/0!
P78344	*EIF4G2*	4.41	1	4	7	102.3	#DIV/0!
P26196	*DDX6*	15.11	5	5	10	54.4	#DIV/0!

Q00059	*TFAM*	8.94	1	2	4	29.1	#DIV/0!
Q9Y266	*NUDC*	8.16	1	2	4	38.2	#DIV/0!
Q96PK6	*RBM14*	5.53	1	3	6	69.4	#DIV/0!
Q15029	*EFTUD2*	4.12	1	3	6	109.4	#DIV/0!
Q7Z2W4	*ZC3HAV1*	3.99	2	4	6	101.4	#DIV/0!
O00148	*DDX39*	3.98	2	2	5	49.1	#DIV/0!
Q52LJ0	*FAM98B*	19.39	1	3	7	37.2	#DIV/0!
Q7L2H7	*EIF3M*	8.56	1	2	6	42.5	#DIV/0!
P09543	*CNP*	4.75	1	2	4	47.5	#DIV/0!
Q96FJ2	*DYNLL2*	26.97	1	1	4	10.3	#DIV/0!
Q9Y295	*DRG1*	11.99	1	3	5	40.5	#DIV/0!
Q96DH6	*MSI2*	14.63	2	3	6	35.2	#DIV/0!
P08621	*SNRNP70*	6.41	1	2	4	51.5	#DIV/0!
Q6P1J9	*CDC73*	3.58	1	2	4	60.5	#DIV/0!
Q13418	*ILK*	3.32	1	2	4	51.4	#DIV/0!
O75569	*PRKRA*	12.14	1	3	7	34.4	#DIV/0!
Q01081	*U2AF1*	13.33	1	2	4	27.9	#DIV/0!
Q9NX05	*FAM120C*	4.29	2	3	4	120.5	#DIV/0!
P08237	*PFKM*	2.31	3	2	4	85.1	#DIV/0!
O95782	*AP2A1*	2.97	2	3	5	107.5	#DIV/0!
Q9BQ70	*TCF25*	2.51	1	2	4	76.6	#DIV/0!
Q8NCA5	*FAM98A*	6.36	1	1	4	55.4	#DIV/0!
Q96I24	*FUBP3*	3.85	1	2	4	61.6	#DIV/0!
Q08945	*SSRP1*	2.68	1	2	4	81.0	#DIV/0!
P78347	*GTF2I*	2.10	1	2	4	112.3	#DIV/0!
Q99439	*CNN2*	9.71	1	2	4	33.7	#DIV/0!
P10644	*PRKAR1A*	7.61	1	2	4	43.0	#DIV/0!
Q01780	*EXOSC10*	2.71	1	2	4	100.8	#DIV/0!
Q86W92	*PPFIBP1*	1.98	1	2	4	114.0	#DIV/0!
P11908	*PRPS2*	10.38	3	2	4	34.7	#DIV/0!
Q9Y2T2	*AP3M1*	5.26	2	2	4	46.9	#DIV/0!
P13010	*XRCC5*	63.80	2	42	145	82.7	477.12
P54821	*PRRX1*	66.53	17	24	210	27.3	463.74
P12956	*XRCC6*	56.98	1	35	123	69.8	269.16
Q14444	*CAPRIN1*	18.76	1	8	22	78.3	45.65
P09874	*PARP1*	28.80	1	22	42	113.0	15.67
Q9NUQ6	*SPATS2L*	18.82	1	10	21	61.7	12.36
Q13283	*G3BP1*	31.55	1	10	35	52.1	11.75
Q8NC51	*SERBP1*	32.35	1	11	27	44.9	11.50
Q13310	*PABPC4*	25.31	1	6	41	70.7	10.65
Q08211	*DHX9*	12.13	1	14	29	140.9	9.76
Q14011	*CIRBP*	7.56	1	2	6	18.6	9.67
Q92900	*UPF1*	6.82	1	7	14	124.3	9.46

P11940	*PABPC1*	43.87	5	16	72	70.6	9.10
P67809	*YBX1*	27.16	2	4	22	35.9	8.05
P52701	*MSH6*	11.62	1	14	29	152.7	7.71
Q14152	*EIF3A*	15.48	1	19	40	166.5	6.76
Q15717	*ELAVL1*	27.91	3	8	17	36.1	6.55
P60842	*EIF4A1*	24.88	3	8	16	46.1	6.52
Q12906	*ILF3*	9.06	1	7	20	95.3	6.46
Q9Y6M1	*IGF2BP2*	17.70	3	6	19	66.1	6.12
Q00839	*HNRNPU*	39.88	1	29	101	90.5	6.08
P16989	*CSDA*	10.22	2	1	15	40.1	5.93
Q6PKG0	*LARP1*	7.94	2	7	14	123.4	5.70
O00425	*IGF2BP3*	8.29	3	3	14	63.7	5.66
O95819	*MAP4K4*	6.62	4	6	14	142.0	5.26
**FLAG-PRRX1A (in untreated U2OS)**							
**Accession**	**Gene symbol**	**Coverage %**	**Protein Qscore**	**Unique Peptides**	**Unique Spectra**	**MW [kDa]**	**iBAQ**
P54821	*PRRX1*	61.22	68.64	16	248	27.3	14882622.8
P78527	*PRKDC*	35.95	483.69	128	156	468.8	940687.34
P13010	*XRCC5*	64.62	136.25	35	86	82.7	9428743.89
P12956	*XRCC6*	66.50	146.16	38	67	69.8	13996875.31
**FLAG-PRRX1A (in ETP treated U2OS)**							
**Accession**	**Gene symbol**	**Coverage %**	**Protein Qscore**	**Unique Peptides**	**Unique Spectra**	**MW [kDa]**	**iBAQ**
P54821	*PRRX1*	61.22	57.38	16	272	27.3	9555254.12
P78527	*PRKDC*	39.07	465.62	138	158	468.8	893686.91
P13010	*XRCC5*	63.93	115.88	34	64	82.7	4456944.14
P12956	*XRCC6*	60.76	125.28	36	62	69.8	6550210.98
**FLAG-PRRX1A (in IR treated U2OS)**							
**Accession**	**Gene symbol**	**Coverage %**	**Protein Qscore**	**Unique Peptides**	**Unique Spectra**	**MW [kDa]**	**iBAQ**
P54821	*PRRX1*	60.82	68.73	16	220	27.3	7148908.72
P78527	*PRKDC*	38.71	504.92	135	147	468.8	498294.53
P13010	*XRCC5*	62.98	134.73	34	67	82.7	4613553.26
P12956	*XRCC6*	63.71	142.32	36	63	69.8	6175306.72
Q13426	*XRCC4*	5.06	3.29	1	1	38.3	63305.81

Table S2. The expression levels (FPKM) of NHEJ genes in MEFs

**Gene ID**	**Gene Symbol**	**FPKM**					
		**WT#23**	**WT#25**	**HET#22**	**HET#24**	**KO#32**	**KO#33**
ENSMUSG00000026586	*Prrx1*	60.6857	62.7862	43.7600	39.1996	19.8508	19.9725
ENSMUSG00000043909	*Trp53bp1*	3.8070	3.8430	4.1294	4.0355	5.0898	4.8998
ENSMUSG00000022672	*Prkdc*	1.6222	1.3994	1.6723	1.4898	2.4170	2.0444
ENSMUSG00000022471	*Xrcc6*	6.1030	5.2815	5.6520	4.8618	6.5075	6.9430
ENSMUSG00000026187	*Xrcc5*	7.5375	9.1884	8.6309	8.6261	8.8084	9.3719
ENSMUSG00000026648	*Dclre1c*	1.1195	0.8029	0.7630	1.0335	0.8380	0.8153
ENSMUSG00000021615	*Xrcc4*	1.2518	1.1922	1.3816	1.3871	1.3163	1.1422
ENSMUSG00000049717	*Lig4*	2.9958	3.0751	2.9480	3.0599	2.4115	2.6638
ENSMUSG00000026162	*Nhej1*	1.9081	2.3929	2.1527	1.8419	2.1671	1.6595
ENSMUSG00000030051	*Aplf*	1.3401	1.6125	1.3981	1.7472	1.9719	1.6440
ENSMUSG00000047617	*Paxx*	2.5813	2.1934	2.7362	2.7747	5.3163	3.9850
ENSMUSG00000025218	*Poll*	3.1107	3.6648	3.5402	3.6401	5.8613	5.0644
ENSMUSG00000020474	*Polm*	6.3986	6.8507	5.5082	4.8688	4.9307	5.0403

Table S3. Antibodies and oligonucleotides used in this study

**Antibodies**				
**Name**	**Application / Dilution**	**Source**	**RRID**	**Cat#**
anti-PRRX1	WB (1:3,000); IP (1:100)	customization	N/A	N/A
anti-PRRX1	WB (1:1,000); IF (1:100)	OriGene	AB_2620166	TA803116
anti-Ku70	WB (1:1,000)	Santa Cruz Biotechnology	AB_628454	sc-17789
anti-Ku70	WB (1:5,000);<br>IP (2 μg/sample);<br>ChIP (3 μg/sample);	Proteintech	AB_2218756	10723-1-AP
anti-Ku86	WB (1:3,000)	Santa Cruz Biotechnology	AB_2890940	sc-515736
anti-Ku80	IP (2 μg/sample)	Proteintech	AB_2257509	16389-1-AP
anti-DNA-PKcs	WB (1:1,000)	Invitrogen	AB_11004133	MA5-13404
anti-DNA-PKcs	IP (6 μg/sample)	Santa Cruz Biotechnology	AB_2172845	sc-9051
anti-DNA-PKcs (p-S2056)	WB (1:5,000); IF (1:500)	Abcam	AB_11001004	ab124918
anti-H2A.X (p-S139)	WB (1:2,000); IF (1:500)	ABclonal	AB_2863808	AP0687
anti-H2A.X (p-S139)	ChIP (3 μg/sample)	Sigma-Aldrich	05-636	AB_309864
anti-MYC	WB (1:1,000)	Cell Signaling Technology;<br>Gift from Dr. Hudan Liu	AB_2631168	13987
anti-53BP1	IF (1:200)	Cell Signaling Technology;<br>Gift from Dr. Qiang Chen	AB_10694558	4937
anti-TBK1	WB (1:1,000)	Cell Signaling Technology;<br>Gift from Dr. Junjie Zhang	AB_2255663	3504
anti-TBK1 pS172	WB (1:1,000)	Cell Signaling Technology;<br>Gift from Dr. Junjie Zhang	AB_10693472	5483
anti-IRF3	WB (1:500)	Santa Cruz Biotechnology;<br>Gift from Dr. Shu Li	AB_2264929	sc-9082
anti-IRF3 pS396	WB (1:500)	Cell Signaling Technology;<br>Gift from Dr. Junjie Zhang	AB_823547	4947
anti-STING	WB (1:1,000)	Cell Signaling Technology;<br>Gift from Dr. Shu Li	AB_2732796	13647
anti-STING pS365	WB (1:1,000)	Cell Signaling Technology;<br>Gift from Dr. Junjie Zhang	AB_2799831	72971
anti-β-Actin	WB (1:300,000)	ABclonal	AB_2768234	AC026
anti-β-Tubulin	WB (1:5,000)	Proteintech	AB_2210695	10094-1-AP
anti-Histone H3	WB (1:20,000)	ABclonal	AB_2631273	A2348
anti-DDDDK-Tag (FLAG)	WB (1:10,000)	MBL	AB_11123930	M185-3L
anti-MBP-Tag	WB (1:5,000)	ABclonal	AB_2770406	AE016
anti-GST-Tag	WB (1:5,000)	ABclonal	AB_2771923	AE006
anti-His-Tag	WB (1:5,000)	ABclonal	AB_2728734	AE003
anti-GFP-Tag	WB (1:5,000)	ABclonal	AB_2770402	AE012
anti-mCherry-Tag	WB (1:5,000)	ELK Biotechnology	N/A	EA007

anti-mCherry-Tag	ChIP (4 µg/sample)	ABclonal	AE002	AB_2770407
Rabbit IgG	IP (equal)	ABclonal	AB_2771930	AC005
Mouse IgG	IP (equal)	Santa Cruz Biotechnology	AB_737182	sc-2025
Alexa Fluor 488-conjugated anti-rabbit	IF (1:1,000)	Invitrogen	AB_2576217	A11034
Alexa Fluor 555-conjugated anti-rabbit	IF (1:1,000)	Invitrogen	AB_2535850	A21429
Alexa Fluor 488-conjugated anti-mouse	IF (1:1,000)	Invitrogen	AB_2534088	A11029
Alexa Fluor 488-conjugated Streptavidin	IF (1:1,000)	Invitrogen	AB_2315383	S32354
anti-mouse IgG (H+L)-HRP	WB (1:10,000)	Jackson ImmunoResearch	AB_10015289	115-035-003
anti-rabbit IgG (H+L)-HRP	WB (1:10,000)	Jackson ImmunoResearch	AB_2313567	111-035-003
anti-mouse IgG (L)-HRP	WB (1:10,000)	Jackson ImmunoResearch	AB_2338512	115-035-174
anti-rabbit IgG (L)-HRP	WB (1:10,000)	Jackson ImmunoResearch	AB_2339146	211-002-171

Oligonucleotides

Name	Sequence (5' to 3')	Purpose	Source
Prrx1-KO-F1	AGCTCCTGCATCCACCAATTAG	genotyping	This paper
Prrx1-KO-R1	TTCTACCACCACTTCCTCTGTGAG	genotyping	This paper
Prrx1-WT-F2	TTCAGGGCATTTGTCGTAACAGGG	genotyping	This paper
EGFP-PA-F	AGCTCAAGCTTCGAATTCATGACCTCCAGCTACGGGCA	EGFP-PRRX1A	This paper
EGFP-PA-R	CCGTCGACTGCAGAATTCTTAGAATCCGTTATGAAGCCCCTC	EGFP-PRRX1A	This paper
EGFP-PB-R	TACCGTCGACTGCAGAATTCTCAGTTGACTGTTGGCACCTGG	EGFP-PRRX1B	This paper
EGFP-N-R	CCGTCGACTGCAGAATTCTTACTTTCTCTTCTTTTTTCTTCTGAGTTCAGCTG	EGFP-N	This paper
EGFP-HD-F	CGAGCTCAAGCTTCGAATTCCAGCGAAGGAATAGGACAACCT	EGFP-HD	This paper
EGFP-HD-R	CCGTCGACTGCAGAATTCTTACTCATTCCTGCGGAACTTGGC	EGFP-ΔC / -HD	This paper
Cherry-PA-F	CCGCTCGAGGGAGACCATGACCTCCAGCTACGGGC	Cherry-PRRX1A	This paper
Cherry-PA-R	CGCGGATCCCTTAGAATCCGTTATGAAGCCCCTCG	Cherry-PRRX1A	This paper
Cherry-PB-R	CGCGGATCCCTCAGTTGACTGTTGGCACCTGG	Cherry-PRRX1B	This paper
Cherry-delC-R	CGCGGATCCTTACTCATTCCTGCGGAACTTGGC	Cherry-ΔC	This paper
pHAGE-PA-XhoI-F	CCGCTCGAGATGACCTCCAGCTACGGGCA	PRRX1A (zsgreen / puro)	This paper
pHAGE-PA-BamHI-R	CGCGGATCCTTAGAATCCGTTATGAAGCCCCTCGT	PRRX1A (zsgreen / puro)	This paper
pHAGE-PB-R	CGCGGATCCTCAGTTGACTGTTGGCACCTGG	PRRX1B	This paper

		(zsgreen / puro)	
pHAGE-FLAG-F	CCGGTGCCACCATGGATTACAAGGATGACGATAAGTTC	Kozak + FLAG tag	This paper
pHAGE-FLAG-R	CTCGAGAACTTATCGTCATCCTTGTAATCCATGGTGGCA	Kozak + FLAG tag	This paper
pET28a-PA-F	GGTCGCGGATCCGAATTCATGACCTCCAGCTACGGG	His-PRRX1A	This paper
pET28a-PA-R	CGACGGAGCTCGAATTCTTAGAATCCGTTATGAAGCCC	His-PRRX1A	This paper
pET28a-PB-R	TCGACGGAGCTCGAATTCTCAGTTGACTGTTGGCACCTG	His-PRRX1B	This paper
pMAL-c2X-PA-F	GAGGGAAGGATTTCAGAATTCATGACCTCCAGCTACGGG	MBP-PRRX1A	This paper
pMAL-c2X-PA-R	TCTAGAGGATCCGAATTCTTAGAATCCGTTATGAAGCCCC	MBP-PRRX1A	This paper
pMAL-c2X-PB-R	CTCTAGAGGATCCGAATTCTCAGTTGACTGTTGGCACCTG	MBP-PRRX1B	This paper
pMAL-c2X-del200-R	GACTCTAGAGGATCCGAATTCCTAGTACGGAGACGCTGTCCCCC	MBP-ΔC18	This paper
pMAL-c2X-N-R	ACTCTAGAGGATCCGAATTCCTACTTTCTCTTCTTTTTTTCTTCTGAGTT	MBP-N	This paper
pMAL-c2X-HD-F	GATCGAGGGAAGGATTTCAGAATTCCAGCGAAGGAATAGGACAACCT	MBP-ΔN / -HD	This paper
pMAL-c2X-HD-R	AGGTCGACTCTAGAGGATCCGAATTCTTACTCATTCCTGCGGAACTTGGC	MBP-ΔC / -HD	This paper
PRRX1-144A-F	GTGCAGGTGTGGTTTCAGGCCCGAAGAGCC	N144A mut	This paper
PRRX1-144A-R	GCCTGAAACCACACCTGCACTCTCGCCTCG	N144A mut	This paper
PRRX1-145Q-F	GTGTGGTTTCAGAACCAAAGAGCCAAGTTC	R145Q mut	This paper
PRRX1-145Q-R	CTTTGGTTCTGAAACCACACCTGCACTCTC	R145Q mut	This paper
PRRX1-145A-F	GTGTGGTTTCAGAACGCAAGAGCCAAGTTC	R145A mut	This paper
PRRX1-145A-R	CTTGCGTTCTGAAACCACACCTGCACTCTC	R145A mut	This paper
PRRX1-146A-F	GTGTGGTTTCAGAACCGAGCAGCCAAGTTC	R146A mut	This paper
PRRX1-146A-R	GCTCGGTTCTGAAACCACACCTGCACTCTC	R146A mut	This paper
NRR-AAA-F	GTGCAGGTGTGGTTTCAGGCCGCAGCAGCCAAG	NRR^{mut}	This paper
NRR-AAA-R	GGCTGCTGCGGCCTGAAACCACACCTGCACTCTCGC	NRR^{mut}	This paper
107A-F	CAGCTGGCGGCTTTGGAGCGTGTCTTTGAGC	Q107A mutant	This paper
107A-R	CAAAGCCGCCAGCTGGCTGCTATTGAAGG	Q107A mutant	This paper
107A/110A-F	TGGCGGCTTTGGCGCGTGTCTTTGAGCG	Q107A/E110A mutant	This paper
107A/110A-R	CGCCAAAGCCGCCAGCTGGCTGCTATTG	Q107A/E110A mutant	This paper
110A/111A-F	GCTTTGGCGGCTGTCTTTGAGCGGACACAC	E110A/R111A mutant	This paper
110A/111A-R	AAGACAGCCGCCAAAGCCTGCAGCTGGCTGCT	E110A/R111A mutant	This paper

EGFP-Ku70-F	GCCGGACTCAGATCTCGAGCATGTCAGGGTGGGAGTC ATATTAC	EGFP-Ku70	This paper
EGFP-Ku70-R	ATTCGAAGCTTGAGCTCGAGTCAGTCCTGGAAGTGCTT GGTGA	EGFP-Ku70	This paper
pGEX-4T1-Ku80-F	ATCTGGTTCCGCGTGGATCCATGGTGCGGTCGGGGAA T	GST-Ku80	This paper
pGEX-4T1-Ku80-R	GGGAATTCCGGGGATCCCTATATCATGTCCAATAAATCG TC	GST-Ku80	This paper
pGEX-4T1-Ku70-F	TGGTTCCGCGTGGATCCATGTCAGGGTGGGAGTCATAT	GST-Ku70	This paper
pGEX-4T1-Ku70-R	CCGGGAATTCCGGGGATCCTCAGTCCTGGAAGTGCTT G	GST-Ku70	This paper
4T1-vWA-R	TCACGATGCGGCCGCTCGAGCTAGAGTGCTCGCTTCC TGGTC	GST-Ku70-vWA	This paper
4T1-Core-F	ATCTGGTTCCGCGTGGATCCAGCAGGTTAAAGCTGAA GCTCAACA	GST-Ku70-Core	This paper
4T1-Core-R	TCACGATGCGGCCGCTCGAGCTATGGGTAAACAAGCT CCTTAA	GST-Ku70-Core	This paper
4T1-SAP-F	ATCTGGTTCCGCGTGGATCCCCAGATTACAATCCTGAA GGGA	GST-Ku70-SAP	This paper
4T1-delSAP-R	TCACGATGCGGCCGCTCGAGCTACGTACCCTTGCTGA TGTGG	GST-Ku70 ΔSAP	This paper
Prrx1-qP-F	GACACCCCTCAGCAGGACAA	qPCR	This paper
Prrx1-qP-R	TGAAACCACACCTGCACTCT	qPCR	This paper
Prkdc-qP-F	AGCCATTGCTATTCGCGGATA	qPCR	This paper
Prkdc-qP-R	CGGAACCGTGTCAAGGTAAAG	qPCR	This paper
Xrcc6-qP-F	AGAAGCACTTCCGAGACACG	qPCR	This paper
Xrcc6-qP-R	TCGTCTTCATTGGTGAACAGC	qPCR	This paper
Gapdh-qP-F	TCGGTGTGAACGGATTTG	qPCR	This paper
Gapdh-qP-R	GGTCTCGCTCCTGGAAGA	qPCR	This paper
PRRX1-qP-F	AGCAGCGAAGGAATAGGACAA	qPCR	This paper
PRRX1-qP-R	GGAACTTGGCTCTTCGGTTCT	qPCR	This paper
PRKDC-qP-F	TGAACACCATGTCCCAAGAGG	qPCR	This paper
PRKDC-qP-R	CAGTACGATTAGCGCCCTTATACA	qPCR	This paper
XRCC6-qP-F	CTGTTCACCAATGAAGACAACC	qPCR	This paper
XRCC6-qP-R	GAACAAGGATATGTCAAAGCCC	qPCR	This paper
ACTB-qP-F	CTCTTCCAGCCTTCCTTCCT	qPCR	This paper
ACTB-qP-R	AGCACTGTGTTGGCGTACAG	qPCR	This paper
DSB-F1	TGGTGAGCAAGGGCGAGGAG	ChIP-qPCR	This paper
DSB-R1	TCGTGCTGCTTCATGTGGTCG	ChIP-qPCR	This paper
Control-F1	CTGGCTGAGTCCTCCCCTG	ChIP-qPCR	This paper
Control-R1	CACTCTCCCACTCCCTAGACTGG	ChIP-qPCR	This paper
Scramble	CCTAAGGTTAAGTCGCCCTCG	shRNA control	This paper
shPRRX1#2	GCTGATACATCTGATCTATCA	shRNA target	This paper

shPRRX1#3	GAGAGCCATGCTAGCCAATAA	shRNA target	This paper
shKu70#1	GTCAGGGTGGGAGTCATATTA	shRNA target	This paper
shKu70#3	GCCCTAAGTTTGTACTATATA	shRNA target	This paper
Oligo#1-S	AAGATGTTGTTTACACGAGGGCTCGAGCCCTCGTGTAA ACAACATCTTTTTTTG (3' Biotin)	DNA pulldown	This paper
Oligo#1-A	CAAAAAAAGATGTTGTTTACACGAGGGCTCGAGCCCTC GTGTAAACAACATCTT	DNA pulldown & MST	This paper
Oligo#2-S	GAGCCCTCGTGTAAACAACATCTTTTTTTG (3' Biotin)	DNA pulldown	This paper
Oligo#2-A	CAAAAAAAGATGTTGTTTACACGAGGGCTC	DNA pulldown	This paper
Oligo#3-S	AAGATGTTGTTTACACGAGGGCTCGAGCCCTCGTGTAA ACAACATCTTTTTTTG (3' 6-FAM)	MST	This paper
Oligo#4-A	CAAAAAAAGATGTTGTTTACACGAGGGCTCGAGCCCTC GTGTAAACAACAT	MST	This paper
Oligo#5-A	CAAAAAAAGATGTTGTTTACACGAGGGCTCGAGCCCTC GTGTAAACAA	MST	This paper
Oligo#6-A	CAAAAAAAGATGTTGTTTACACGAGGGCTC	MST	This paper
Note: All the above DNA oligonucleotides were synthesized by Sangon Biotech			
